# Behavioral Senescence and Aging-Related Changes in Motor Neurons and Brain Neuromodulator Levels Are Ameliorated by Lifespan-Extending Reproductive Dormancy in *Drosophila*

**DOI:** 10.3389/fncel.2017.00111

**Published:** 2017-04-20

**Authors:** Sifang Liao, Susan Broughton, Dick R. Nässel

**Affiliations:** ^1^Department of Zoology, Stockholm UniversityStockholm, Sweden; ^2^Division of Biomedical and Life Sciences, Faculty of Health and Medicine, Lancaster UniversityLancaster, UK

**Keywords:** diapause, aging, sleep, negative geotaxis, walking behavior, insulin signaling, neuropeptides, dopamine

## Abstract

The lifespan of *Drosophila* *melanogaster* can be extended substantially by inducing reproductive dormancy (also known as diapause) by lowered temperature and short days. This increase of longevity is accompanied by lowered metabolism and increased stress tolerance. We ask here whether behavioral senescence is ameliorated during adult dormancy. To study this we kept flies for seven or more weeks in normal rearing conditions or in diapause conditions and compared to 1-week-old flies in different behavioral assays of sleep, negative geotaxis and exploratory walking. We found that the senescence of geotaxis and locomotor behavior seen under normal rearing conditions was negligible in flies kept in dormancy. The normal senescence of rhythmic activity and sleep patterns during the daytime was also reduced by adult dormancy. Investigating the morphology of specific neuromuscular junctions (NMJs), we found that changes normally seen with aging do not take place in dormant flies. To monitor age-associated changes in neuronal circuits regulating activity rhythms, sleep and walking behavior we applied antisera to tyrosine hydroxylase (TH), serotonin and several neuropeptides to examine changes in expression levels and neuron morphology. In most neuron types the levels of stored neuromodulators decreased during normal aging, but not in diapause treated flies. No signs of neurodegeneration were seen in either condition. Our data suggest that age-related changes in motor neurons could be the cause of part of the behavioral senescence and that this is ameliorated by reproductive diapause. Earlier studies established a link between age-associated decreases in neuromodulator levels and behavioral decline that could be rescued by overexpression of neuromodulator. Thus, it is likely that the retained levels of neuromodulators in dormant flies alleviate behavioral senescence.

## Introduction

Organismal lifespan is dependent on genetic and environmental factors such as diet and exposure to xenobiotics or other stressors, and can be extended experimentally in several model organisms via the manipulation of nutrient sensing and stress response signaling pathways (reviewed in Guarente and Kenyon, 2000; Kenyon, 2005; Fontana et al., 2010). For instance, dietary restriction and the genetic down regulation of the nutrient-sensing insulin-IGF-like signaling (IIS) and target of rapamycin (TOR) networks are the most well studied means of extending lifespan in model organisms (Guarente and Kenyon, 2000; Clancy et al., 2001; Tatar et al., 2001; Broughton et al., 2005; Jones et al., 2009; Fontana et al., 2010). In *Drosophila*, several genetic manipulations of the IIS pathway are known to extend lifespan, including mutations in the insulin receptor (InR), *Drosophila* insulin-like peptide ligands (DILPs) and the InR substrate (*chico*; Clancy et al., 2001; Tatar et al., 2001; Partridge et al., 2011). There is also some evidence that reduced IIS attenuates some forms of age-related health declines and functional senescence (Martin and Grotewiel, 2006; Rhodenizer et al., 2008; Selman et al., 2008). However, the relationship between extended lifespan and health span is not clear because some forms of functional declines are not always ameliorated in long-lived animals (Cook-Wiens and Grotewiel, 2002; Burger and Promislow, 2006; Bhandari et al., 2007; Burger et al., 2008). In particular, the effect of increased lifespan on aging-related decline of neuronal function in the brain and associated behavioral and cognitive senescence is poorly understood. This is an important issue in modern society where life expectancy increases and thereby the incidence of various forms of dementia becomes increasingly problematic (Price et al., 1998; Metaxakis et al., 2014).

*Drosophila* with its short lifespan of around 9–10 weeks, depending on strain, and genetic tractability is an excellent model to study the relationship between lifespan extension and brain and behavior senescence. Here we explore a novel approach to extend adult lifespan for analysis of the effects of aging on brain function in *Drosophila*: induction of adult reproductive dormancy by lowered temperature and shortened day length (Tatar and Yin, 2001; Kubrak et al., 2014; Kučerová et al., 2016). Many insect species respond to seasonal and other environmental changes by entering dormancy, also known as diapause, which postpones development and/or reproduction (Tatar and Yin, 2001; Denlinger, 2002; Denlinger and Armbruster, 2014). In *Drosophila* exposure to low temperature and short days just after adult eclosion results in arrested maturation of reproductive organs and changes in hormonal signaling that reduces feeding, alters metabolism and energy storage, increases stress resistance, and extends lifespan drastically (Kubrak et al., 2014; Kučerová et al., 2016). The dormant flies display decreased systemic insulin signaling, and a genome-wide transcriptomics analysis showed that many genes affected by dormancy are also part of networks regulating aging and lifespan (Kučerová et al., 2016). However, it is not known how this extended lifespan affects aging of tissues, including the brain, and whether behavioral senescence is affected by dormancy. Thus, we ask here whether the lifespan extension caused by adult reproductive dormancy is accompanied by a slowed aging of the central nervous system. This may be anticipated, since it was shown that flies kept for 9 weeks in dormancy and then brought back to non-diapause conditions live on for an additional normal lifespan (Tatar and Yin, 2001). Itis important to mention that lifespan extension caused by dormancy in *Drosophila* is not a simple effect of reduced rate of living due to the low temperature (Tatar and Yin, 2001). These authors showed that senescence is the same in flies that were dormant for 3, 6 and 9 weeks, and also that dormancy was reversed by administration of juvenile hormone, suggesting that slowed aging during dormancy is dependent on endocrine regulation and not low temperature *per se* (Tatar and Yin, 2001).

The *Drosophila* brain has been explored extensively to model neurodegenerative diseases (Chan and Bonini, 2000; Lu and Vogel, 2009). However, in contrast to mammals, the brain of *Drosophila* does not display obvious histological signs of neurodegeneration or apoptosis with increasing age, unless genetically manipulated or exposed to oxidative stress (see Chan and Bonini, 2000; White et al., 2010; Mohan et al., 2014). Only more subtle signs of aging have been revealed in the fly: central neurons do display some ultrastructural changes (Martín-Peña et al., 2006) and certain structural and molecular features were shown to be affected in axon terminations of motor neurons (Beramendi et al., 2007; Wagner et al., 2015). On the other hand, many behaviors, including learning and memory, are known to deteriorate over the lifespan (Grotewiel et al., 2005; Tonoki and Davis, 2012; Yamazaki et al., 2014) suggesting that aging induces deleterious effects on neurons and neuronal circuits in the *Drosophila* brain. One aim of this study is to reveal such aging-induced changes in brain neurons and try to relate them to behavioral senescence. Furthermore, we ask whether dormancy ameliorates the functional decline of neurons.

Here we performed behavioral assays in flies over seven or more weeks of normal aging and in specimens that had been kept in diapause conditions for the same duration. We recorded rhythmic activity and sleep patterns, complex walking behavior, and simple reflex climbing behavior (negative geotaxis), all known to change with age (Gargano et al., 2005; Koh et al., 2006; Umezaki et al., 2012; Ismail et al., 2015). In the peripheral nervous system it has been shown that the morphology of the axon terminations of motor neurons changes with age (Beramendi et al., 2007; Wagner et al., 2015). Hence, we analyzed expression of proteins in and morphology of the axon terminations of abdominal motor neurons in our experimental flies. To assay aging of brain neurons during dormancy and control conditions, we monitored neuronal expression of neuromodulators such as the monoamines dopamine and serotonin, and several neuropeptides since transcript levels of dopamine signaling components and many of the peptides are affected by dormancy (Kučerová et al., 2016). We found that, compared to normally aging flies, the ones kept under diapause conditions for 7–8 weeks display strongly reduced behavioral senescence, and the expression levels of monoamines and some of the neuropeptides analyzed decrease during normal aging, but not in dormant flies. Some of these neuromodulators are associated with the mushroom bodies and central complex, brain centers that regulate behaviors shown to deteriorate with age (Grotewiel et al., 2005; Koh et al., 2006; Tonoki and Davis, 2012). Another prominent example is pigment-dispersing factor (PDF), which is an important neuropeptide in a set of clock neurons that participate in regulation of activity rhythms and sleep (Helfrich-Förster et al., 2007; Nitabach and Taghert, 2008); this peptide was seen to decrease in normally aging flies, but not in dormant ones.

In conclusion we found that reproductive dormancy not only extends lifespan, but also slows down behavioral senescence and ameliorates aging-associated changes in motor neurons and reduction of neuromodulator levels in neurons of the *Drosophila* brain.

## Materials and Methods

### Fly Stocks and Maintenance

*Drosophila melanogaster* of the Canton S strain obtained from Bloomington *Drosophila* Stock Center (BDSC, Bloomington, IN, USA) were used in the experiments. This strain was selected since it is commonly used in *Drosophila* diapause/dormancy studies due to its relatively high diapause propensity (Saunders et al., 1989; Tatar and Yin, 2001; Kubrak et al., 2014). Thus, dormancy induction is likely to be maximally efficient in our experiments. Artificial food medium containing 100 g/L sucrose, 50 g/L yeast, 12 g/L agar, 3 ml/L propionic acid and 3 g/L nipagin was used for raising and keeping the flies. For diapause experiments virgin female flies were collected within 4 h after eclosion, put under CO_2_ anesthesia, and 10–15 flies were transferred to each plastic vial on food medium. Following earlier studies (Saunders et al., 1989; Kubrak et al., 2014), the flies were placed in incubators either under normal conditions with 25°C and 12 h light:12 h dark (12L:12D), or under diapause conditions with 11°C and 10L:14D. It can be noted that dormancy can also be induced at 11°C and 12L:12D (Liu et al., 2016a), but with days longer than 16 h it cannot (Saunders et al., 1990). Flies kept under normal conditions were changed to fresh food twice a week during daytime, and the number of dead flies was counted at each transfer to fresh food. The flies reared under diapause conditions were transferred to fresh food only once, after 4 weeks (performed swiftly in an 18°C room).

### Negative Geotaxis Assay

Negative geotaxis of female flies was measured using the Rapid iterative negative geotaxis (RING) assay, as described previously (Gargano et al., 2005). Briefly, flies were placed into 35 mL plastic vials kept upright, then tapped to the bottom of the tube, and flies climbing over 5 cm in the tube after 10 s were scored. For each replicate three trials were performed, separated by 1 min intervals. The percentage of flies climbing over 5 cm within 10 s was calculated. Flies of different ages maintained under normal conditions, diapause conditions and returned back to normal conditions after 3 weeks of diapause (diapause recovery) were used for testing. Before testing, the flies taken from diapause conditions were allowed to recover for 24 h under control conditions.

### Locomotor Activity and Sleep Assays

To assess the locomotor activity rhythm, we used flies that had been kept under control conditions for 1 or 6 weeks, or under diapause conditions for 5 weeks plus 1 week recovery under normal conditions. Flies were placed in glass tubes containing 5% sucrose in 2% aqueous agar plugged with paraffin wax; small pieces of sponge were used for the other end of the tube. Locomotor activity was monitored under 12L:12D for 7 days using the *Drosophila* Activity Monitoring (DAM) System (Trikinetics, Waltham, MA, USA). The data collected from days 3–6 were used for analysis. Number of sleep bouts was calculated using BeFLY! Analysis Tools v7.23 (Ed Green) in Excel.

### Exploratory Walking Recording

Procedures for lifespan studies are as described in Clancy et al. (2001). Lifespan was measured in virgin female flies, 10 flies/vial under normal and diapause conditions. Flies were transferred to new food three times a week and deaths were scored 4–5 times in every 7 days. Walking behavior of individual female flies was measured as described in Ismail et al. (2015). Female flies, generated and maintained in the same way as flies for survival analysis, were aspirated into individual 4 cm diameter arenas, allowed to rest for 1 min and then were videoed for 15 min. Videos were analyzed using Ethovision XT video tracking software (Noldus). Walking behavior was measured in this way at 1, 2, 4, 7, 8 and 11 weeks of age. The statistical analysis is presented in a specific section below.

### Antisera and Immunocytochemistry

All flies were sampled at the same time in the morning (about 10:00), except flies to be used for PDF immunolabeling, which were collected 2 h after lights on in the morning. The opened fly heads were fixed for 3 h in ice-cold 4% paraformaldehyde (4% PFA) in 0.1 M phosphate buffered saline (PBS), and subsequently rinsed in PBS three times for 1 h. The brain was dissected out in PBS. For staining of the neuromuscular junction (NMJ) fly abdomens were dissected in PBS to expose the ventral abdominal muscles and fixed in PFA as above. The dissected adult tissues were used for whole mount immunocytochemistry and were incubated with primary antiserum for 48 h at 4°C and 30 min at room temperature, rinsed with PBS with 0.25% Triton-X 100 (PBS-Tx) four times, then incubated with secondary antibody for 48 h at 4°C. After a thorough wash in PBS-Tx tissues were mounted in 80% glycerol in 0.01 M PBS.

The following primary antisera were used: rabbit anti-DILP2 (1:2000) from J. Veenstra, Bordeaux, France (Veenstra et al., 2008), rabbit anti-pigment-dispersing hormone (1:3000) from H. Dircksen, Stockholm, Sweden (Dircksen et al., 1987), rabbit anti-FMRFamide (code 117:I; 1:4000) from C. Grimmelikhuijzen, Copenhagen, Denmark, mouse anti-serotonin (Clone 5HT-H209; 1:80) from Dako, Copenhagen, Denmark, mouse anti-tyrosine hydroxylase (TH) (1:200) from Incstar Corp., Stillwater, MO, USA (Nässel and Elekes, 1992), rabbit anti-horseradish peroxidase (HRP; #323-005-021; 1:500) from Jackson ImmunoResearch, West Grove, PA, USA (Sun and Salvaterra, 1995), rabbit anti-sNPF precursor (1:4000) from J.A. Veenstra (Johard et al., 2008), rabbit anti-corazonin (1:2000) from J.A. Veenstra (Veenstra and Davis, 1993), mouse monoclonal anti-Bruchpilot (nc82; 1:50) from Developmental Studies Hybridoma Bank, University of Iowa (DSHB), rabbit anti-discs large (Dlg; 1:2000) from DSHB (Parnas et al., 2001).

The following secondary antisera were used: goat anti-rabbit Alexa 546 antiserum, and goat anti-mouse Alexa 488 antiserum, both from Invitrogen (both used at a dilution of 1:1000).

### Imaging and Measurements

The brain and the abdominal hemisegments A2 and A3 were scanned with a Zeiss LSM 780 confocal microscope (Jena, Germany) using 20×, 40× oil or 63× oil immersion objectives. Identical laser intensity and scan settings were used for all images of each experiment, including all groups of different ages.

The staining intensity of both cell bodies, or the structure of interest, and a standardized tissue background was quantified using ImageJ 1.40 from NIH, Bethesda, MD, USA^1^ as described in Liu et al. (2016b). Mean staining intensity of the structure was determined by subtracting the background intensity of the tissue.

The branch length and the diameter (longest diameter) of boutons of the NMJs were measured manually. In aging flies, it is difficult to distinguish individual boutons because of the increased size and merging with neighboring boutons, also the intensity decreased in aged flies. Thus, for these we only measured the diameter of boutons that could be clearly distinguished and also used the postsynaptic discs large immunolabeling to aid in defining boutons (see Supplementary Figure S1B). The number of bruchpilot punctae were counted in axonal segments with a defined length and presented as punctae per μm.

For the small ventral lateral clock neurons (s-LN_v_), we used a square of 10 μm × 10 μm, and placed it on the central part of the s-LN_v_s to measure intensity as described in Wülbeck et al. (2008). For intensity measurements of the dorsal projection (DP) of these neurons, we followed (Wülbeck et al., 2008; Umezaki et al., 2012) and used a rectangle of 2.5 μm × 15 μm and placed it on the region before the main branch start. Cell body sizes were measured after immunolabeling as detailed in Liu et al. (2016b). In that study it was demonstrated that using antisera to peptides and serotonin provides a reliable measurement of cell body size as compared to Gal4-driven green fluorescent protein.

### Statistics

For statistics and graphs we used GraphPad Prism version 6.00 (La Jolla, CA, USA^2^). Results are presented as means ± SEM. Data were checked with Shapiro-Wilk normality test, then one or two-way analysis of variance (ANOVA) was performed followed by Tukey’s multiple comparisons test. Student’s *t*-test was used in some cases.

Specifically for walking and lifespan (in Figure 3) statistical analyses were performed using JMP (version 8) software (SAS Institute). Lifespan data were subjected to survival analysis (Log Rank tests with Mantel-Cox post test) and presented as survival curves. Walking data were tested for normality using the Shapiro-Wilk W test, and appropriately transformed where necessary. ANOVA were performed and planned comparisons of means were made using Tukey-Kramer HSD, *p* < 0.05. Data are presented as means of raw data ± SEM. Graphs were made in GraphPad Prism.

**Figure 1:**
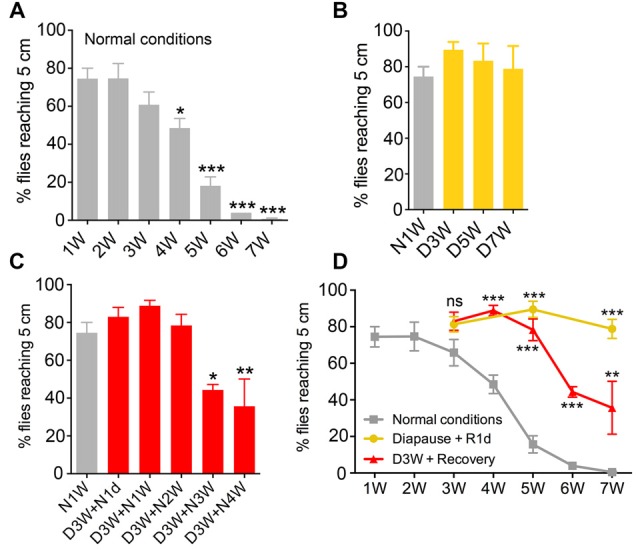
**Negative geotaxis is impaired by aging under normal conditions, but senescence is negligible under diapause conditions**. The percentage of flies climbing over 5 cm within 10 s was recorded at different time points from flies raised under different conditions (N, non-diapause; D, diapause conditions). **(A)** Effect of age on negative geotaxis in flies reared under non-diapause conditions over 7 weeks (1W–7W). After 4 weeks significant impairment is seen. **(B)** Flies kept under diapause conditions (D) do not display significant changes in climbing over 7 weeks compared to 1-week-old flies kept under normal conditions (N1W). **(C)** When flies taken from 3 weeks of dormancy are allowed to recover for different periods (D3W + N1W to D3W + N4W) a decline in climbing ability is seen after 3 weeks recovery. **(D)** Comparison of negative geotaxis responses over time in the three groups of flies: diapause conditions (recovered for 1 day; Diapause + R1d), non-diapause conditions (normal), and flies recovering after 3 weeks of dormancy. The comparisons shown in **(D)** are to normal conditions at similar time points. Data are presented as means ± SEM, *n* = 3–6 independent replicates with 7–14 flies in each replicate. In all experiments flies were assessed in three consecutive trials separated by 60 s of rest (*p < 0.05, **p < 0.01, ***p < 0.001, as assessed by one-way analysis of variance (ANOVA) followed by Tukey’s multiple comparisons or unpaired Students’ *t*-test).

**Figure 2:**
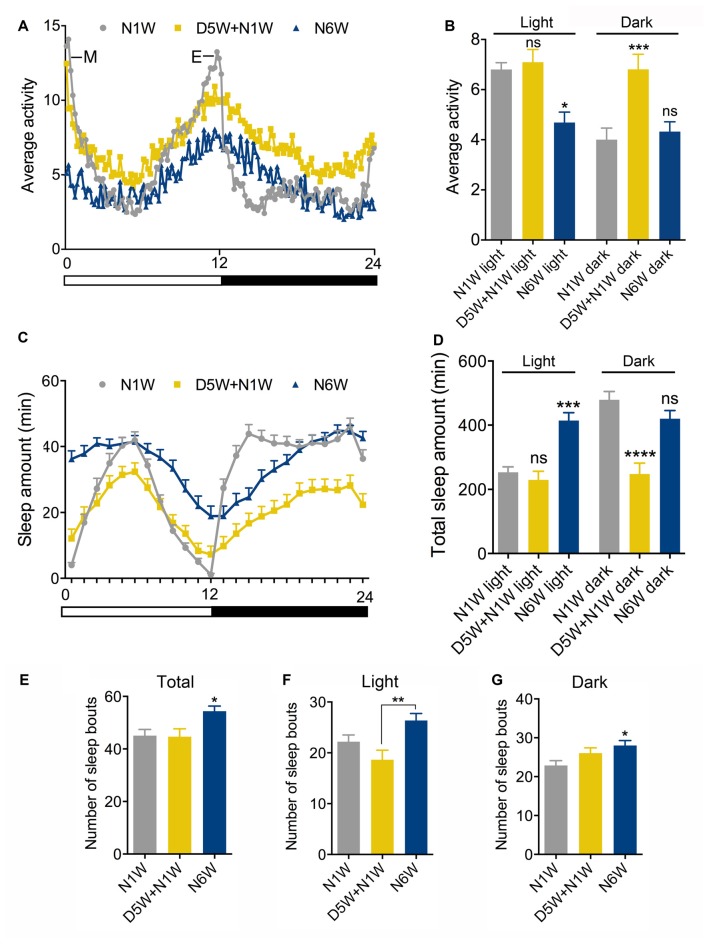
**Diapause conditions diminish senescence of locomotor activity rhythm and sleep patterns**. Locomotor activity rhythms and sleep pattern was monitored in female flies of different ages in normal 12 h light-12 h dark (12L:12D) cycles. **(A,B)** The average locomotor activity of N1W, D5W + N1W and N6W flies measured over 4 days. Note in **(A)** that morning (M) and evening (E) activity is strongly reduced in normally aged flies (N6W). During light phase, the N6W flies displayed lower average activity than N1W flies, whereas flies kept under diapause conditions (D5W + N1W) behaved like young flies. During the dark phase, the (D5W + N1W) flies display a significant increased average activity compared to N1W and N6W flies. **(C)** Sleep pattern of 1-week-old (N1W) flies, flies kept 5 weeks under diapause conditions and allowed to recover 1 week (D5W + N1W) and flies kept 6 weeks in non-diapause condition (N6W) during light phase from ZT1-ZT12 and dark phase from ZT13-ZT24. Each point represents sleep amount in 1 h. The data shown are average of 4 days of recordings, collected from 3^rd^–6^th^ day in the Trikinetics set-up. **(D)** The total sleep time of N6W flies is longer than N1W flies and D5W + N1W in daytime, whereas D5W + N1W flies sleep less compared to N1W and N6W flies during the dark period. **(E)** Number of sleep bouts shown as means per day (24 h) measured over 4 days. Flies kept for 6 weeks under normal conditions display significantly higher number of sleep bouts than both N1W and flies kept in diapause.** (F)** Mean number of sleep bouts during light period over 4 days. **(G)** Mean number of sleep bouts during dark period over 4 days. Data in graphs are presented as means ± SEM, *n* = 27–28 flies for each treatment from three replicates (*p < 0.05, **p < 0.01, ***p < 0.001, ****p < 0.0001, compared to N1W flies, as assessed by one way ANOVA followed by Tukey’s multiple comparisons). The data collected from day 3–6 were used for analysis.

**Figure 3:**
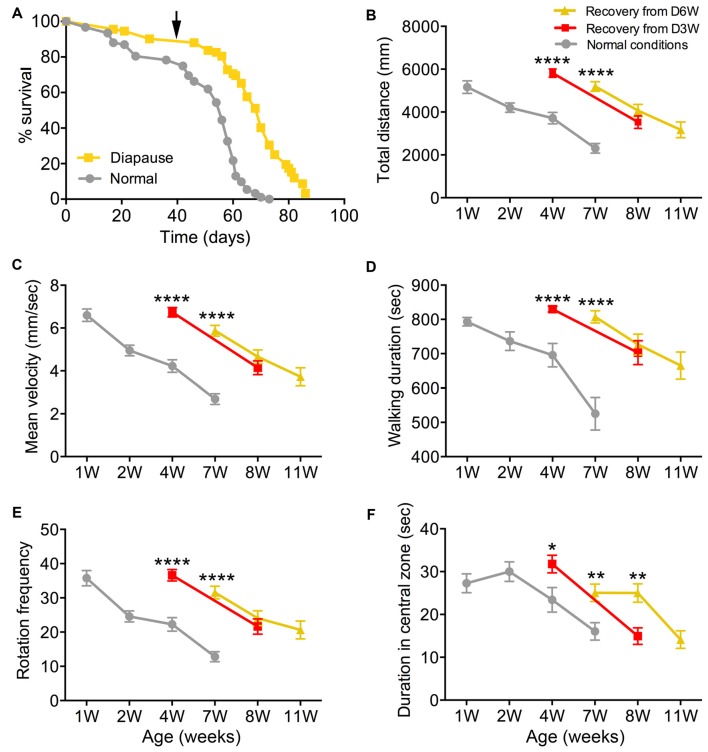
**Diapause conditions delay the onset of exploratory walking senescence. (A)** Survival of female flies under normal and 6 weeks diapause conditions. The arrow indicates the 6-week time point after which the diapause flies (D6W) were transferred to normal conditions. Median lifespans and sample sizes were: Normal conditions = 56 days, *N* = 92; and 6 weeks Diapause conditions = 70 days, *N* = 92. Survival curves were compared using nonparametric log rank tests. Six weeks of diapause treatment resulted in an increase in survival compared to Normal conditions (*P* < 0.0001) with an extension of both median and maximum lifespan. **(B–F)** Exploratory walking senescence for female flies of the indicated conditions run in parallel and under the same conditions as the survival experiment shown in **(A)**. A 3-week diapause condition (D3W) was included in addition to the Normal and D6W conditions. Data are shown as mean value for each walking parameter ± SEM, *N* = 24–44 for the indicated conditions from three replicates. **(B)** Mean distance walked (mm) vs. age. **(C)** Mean velocity (mm/s) v/s age. **(D)** Mean walking duration (s) vs. age. **(E)** Mean frequency of rotations (change in walking direction) vs. age. **(F)** Mean Frequency of Central Zone visits vs. age. Data for each condition were analyzed by ANOVA and age was found to have a significant effect for all conditions (normal, D3W, D6W; *p* < 0.05). Planned comparisons of means were performed using Tukey HSD. For all walking parameters, no significant differences were found between 1 week old normal, 4 week old D3W and 7-week-old D6W flies (*p* > 0.05). At 4 weeks, D3W flies performed significantly better than 4-week-old normal flies. At 7 weeks, D6W flies performed significantly better in all parameters than 7-week-old normal flies. ****Indicates significant difference between diapause treated and normal flies, *p* < 0.0001. **Indicates significant difference between diapause treated and normal flies, *p* < 0.01. *Indicates significant difference between diapause treated and normal flies, *p* < 0.05.

### Quantitative Real-Time PCR (qPCR)

Total RNA was isolated from heads of virgin female flies using Trizol-chloroform (Sigma-Aldrich) from two to three independent biological replicates with 25–30 heads in each replicate. After adjusting the concentration of the RNA by NanoDrop 2000 spectrophotometer (Thermo Scientific), cDNA was prepared using random hexamer primer (Thermo Scientific) and RevertAid reverse transcriptase (Thermo Scientific). The cDNA was then diluted and applied for qPCR using a StepOnePlus^TM^ instrument (Applied Biosystem, USA) and SensiFAST SYBR Hi-ROX Kit (Bioline) as recommended by the manufacturer. The mRNA levels were normalized to rp49 levels in the same samples. Relative expression values were determined by the 2^−∆∆CT^ method (Livak and Schmittgen, 2001). The following sequences of primers were used for qPCR:

*dilp1* F: CGGAAACCACAAACTCTGCG

*dilp1* R :CCCAGCAAGCTTTCACGTTT

*dilp2* F: AGCAAGCCTTTGTCCTTCATCTC

*dilp2* R: ACACCATACTCAGCACCTCGTTG;

*dilp3* F: TGTGTGTATGGCTTCAACGCAATG

*dilp3* R: CACTCAACAGTCTTTCCAGCAGGG;

*dilp5* F: GAGGCACCTTGGGCCTATTC

*dilp5* R: CATGTGGTGAGATTCGG;

*dilp6* F: CCCTTGGCGATGTATTTCCCAACA

*dilp6* R: CCGACTTGCAGCACAAATCGGTTA;

*InR F*: ACTGAACCTCTCGTCAAGGC

*InR R*: GAACCCTCCACGCACTTACA;

*4ebp* F: CCAGGAAGGTTGTCATCTCG

*4ebp* R: CCAGGAGTGGTGGAGTAGAGG;

*Dp110* F: AGTCCACCTCCACA AGTCGAT

Dp110 R: TGTGCAGCGTCA ACTGAAAG;

*Dimm* F: GATGCACAGCCTAAACGA

*Dimm* R: TTTGGCCAGTGTGAGTGT;

*Th* F: GCAAGGCAAATGACGATTCCC

*Th* R: AATCGTTCTTGGTCGGCTGT

*ChAT* F: CCAACCGGATCCGAAAGGAG

*ChAT* R: TTCCATCCTGTATCGCTCGG

*rp49* F: ATCGGTTACGGATCGAACAA

*rp49* R: GACAATCTCCTTGCGCTTCT

## Results

### Dormancy Delays Senescence of Negative Geotaxis

Throughout this study dormancy was induced by placing 4 h old virgin female flies in incubators at 11°C and 10L:14D (on regular food), as performed in earlier work (Saunders et al., 1989; Tatar and Yin, 2001; Kubrak et al., 2014). We refer to this as diapause conditions. Control sibling flies were kept at 25°C and 12L:12D, referred to as normal conditions. We, however, use the term dormancy, and not diapause, for the physiological condition of the flies, since it may be argued that in laboratory strains of *D. melanogaster* (such as Canton S used in this study) dormancy is not induced in advance of aversive environmental conditions, but is actually triggered by them (Tauber et al., 2007). Note that 11°C efficiently induces dormancy also with longer days (up to 16L:8D), suggesting that the low temperature is critical (see Liu et al., 2016a).

RING was used to test the climbing ability of the flies as described earlier (Gargano et al., 2005). Similarly to the previous studies (Gargano et al., 2005; Bhandari et al., 2007), 4-week-old flies reared under normal conditions showed a significant decline in their climbing ability compared to young flies (Figure 1A). The climbing ability declined further in flies that are 5–7 weeks old (Figure 1A). Next, we tested the climbing in flies reared under diapause conditions for 3, 5 and 7 weeks and allowed to recover for 1 day. Interestingly, flies reared under diapause conditions performed as well as the young flies in the negative geotaxis assay (Figure 1B).

We next tested whether this reduction of age-dependent decline in climbing ability of dormant flies is maintained when they are allowed to recover for a longer time. Hence, we raised the flies under diapause conditions for 3 weeks and allowed recovery in normal conditions for 1–4 weeks. These flies displayed a similar trend in age-dependent decline in climbing ability as the flies kept under normal conditions (Figure 1C). When comparing the climbing ability of aging flies, dormant flies and flies that recovered from 3 weeks diapause (Figure 1D), we found that diapause-exposed flies performed significantly better than 5–7 weeks old flies and the recovered flies also showed better climbing ability than similar aged flies reared under normal conditions. Thus, in conclusion, climbing ability declined with aging in flies; however, this decline was negligible if the flies were reared under diapause conditions.

### Dormancy Affects Senescence of Rhythmic Activity and Sleep Patterns

In *Drosophila* behavioral rhythms become weaker and sleep patterns change with age (Koh et al., 2006; Luo et al., 2012; Umezaki et al., 2012). To test the effect of diapause conditions on locomotor rhythm and sleep, we recorded activity using a *Drosophila* activity monitor system in young (1 week), aging (6 weeks) and dormant flies. The latter were placed in diapause conditions for 5 weeks, then transferred to normal conditions for 1 week (D5W + N1W), to entrain the diapausing flies to a 12L:12D cycle and allow them to recover their physiology (see Kubrak et al., 2014) before testing.

As expected, morning and evening activity was strongly reduced in normally aged flies (N6W) compared to young flies (N1W; Figure 2A). Diapause treated flies (D5W + N1W) displayed morning and evening peaks of activity that were similar to young flies, suggesting that dormancy delays the aging related weakening of activity rhythms. Interestingly, the effect of dormancy on activity in the dark and light phases suggests aging and non-aging related effects, respectively. During the light phase, the average locomotor activity decreased in aging flies compared to young flies, but no significant difference was found between dormant and young flies (Figure 2B). In contrast, during the dark phase, average locomotor activity of young and normally aged flies did not change, indicating that, unlike sleep during the light, dark phase sleep does not decline during normal aging. The diapause treated flies displayed a significant increase in dark phase average activity compared to both young and normally aged flies (Figure 2B). Together, these data suggest that diapause treatment both protects against the normal senescence of light phase activity rhythms and has a non-aging-related effect on dark phase sleep. Flies that were taken from diapause conditions were more active than the flies kept in normal conditions for the same time duration due to an aging-independent increase in dark phase activity.

The sleep patterns of young, aging and diapause treated flies are shown in Figure 2C. Similarly to previous studies (Koh et al., 2006; Luo et al., 2012), we found that aging altered the pattern of sleep, and diapause treatment resulted in a different pattern of sleep compared to both young and normally aging flies (Figure 2C). Normally aged flies showed an increase in total day sleep but no change in total night sleep (Figures 2C,D). In addition, during both the day and night, mean number of sleep bouts increased in normally aged flies (Figures 2E–G) compared to young flies and flies kept under diapause conditions. These data indicate that normal aging results in an increase in day sleep duration and an increase in both day and night sleep fragmentation. Diapause treated flies did not show the normal aging related increase in day sleep, and in addition showed a decrease in night total sleep duration (Figures 2C–E) in line with their increased activity during the dark. The effect of diapause treatment on sleep bout number indicates that it protects against aging related changes in day sleep fragmentation but is less protective against night sleep fragmentation (Figures 2E–G). Thus, diapause treatment appears to ameliorate the normal aging related changes in daytime sleep but results in an aging-independent decrease in night sleep duration.

### Dormancy Delays the Onset of Exploratory Walking Senescence

Female Canton-S flies maintained under diapause conditions (11°C, 10:14 LD cycle) for 6 weeks before being transferred to normal conditions (25°C, 12:12 LD cycle) showed, as expected, a dramatically extended median and maximum lifespan compared to flies maintained under normal conditions throughout life (Figure 3A). Exploratory walking was measured in female flies maintained under the same conditions (normal and D6W) at time points throughout life (Figures 3B–F). In addition, females maintained under diapause conditions for 3 weeks (D3W) before being transferred to normal conditions were analyzed. Canton-S females under normal conditions showed declines in all walking parameters with age (Figures 3B–F), which were similar to those seen previously for females in the Dahomey genetic background (Ismail et al., 2015). One week after 3 and 6 weeks of diapause treatment (D3W and D6W, respectively), no significant decline in performance of each walking parameter was seen compared to 1-week-old normal flies (Figures 3B–F). Furthermore, when the D3W and D6W flies were compared to age-matched flies under normal conditions, they performed significantly better. Following the transfer to normal conditions, the D3W and D6W flies showed a normal decline in all walking parameters with age (Figures 3B–F). Thus, dormancy delays the onset of walking senescence with little effect on the subsequent rate of decline seen after reintroduction to normal conditions.

### Dormancy Affects Aspects of Aging of the Neuromuscular Junction

**Figure 4:**
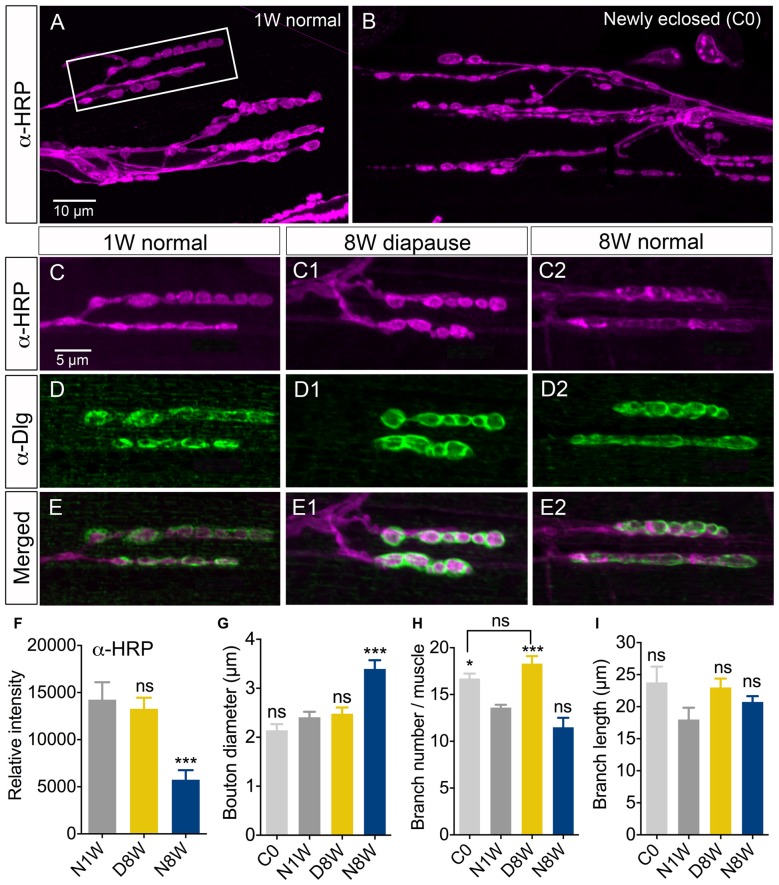
**Senescence of axon terminations in neuromuscular junction (NMJ) is delayed under diapause conditions**. The morphology and horseradish peroxidase (HRP) immunostaining (α-HRP) changed in axon terminations of NMJs in abdominal ventral body wall muscles (VLM) in normally aging flies, but not during diapause conditions. Confocal images of NMJs from the second or third VLM of 1-week old **(A,C–E)**, newly enclosed (C0; **B**) and flies kept for 8 weeks in diapause conditions **(C1–E1)** or 8 weeks flies in normal conditions **(C2–E2)**. NMJs are immunolabeled with antiserum to HRP (α-HRP), a neuronal membrane marker and to discs large (α-Dlg) that labels the subsynaptic reticulum (SSR). **(F)** Relative intensity of α-HRP labeling decreases in flies kept for 8 weeks in normal conditions (N8W), 8 weeks in dormancy (D8W); both compared to 1 week in normal conditions (N1W). **(G)** The diameter of axonal boutons increases only in N8W flies. In Supplementary Figure S1B we show how boutons were measured. **(H)** The number of axon branches is higher in newly enclosed flies (C0) and flies kept 8 weeks in diapause conditions (D8W). **(I)** The branch length is not affected by aging or dormancy. The (α-Dlg) immunolabeling does not change with age or dormancy (not shown). In these experiments 7–10 NMJs of the second or third VLM from 5–7 flies from three different replicates were analyzed (*p < 0.05, ***p < 0.001, as assessed by one-way ANOVA followed by Tukey’s multiple comparisons). In Supplementary Figure S1 overviews of the HRP-labeled NMJ are shown, in Supplementary Figure S1C we quantify Dlg immunolabeling and in Supplementary Figure S2 we show labeling with the synapse protein bruchpilot.s.

Since the age-dependent changes in negative geotaxis are likely to be caused largely by age effects on peripheral tissues such as muscles or NMJ components, we investigated a specific NMJ after exposure to different conditions. We selected the second and third ventral longitudinal muscle (VLM) of the abdominal body wall for analysis since this has been investigated during aging previously (Hebbar et al., 2006; Wagner et al., 2015). NMJs from flies exposed to 8 weeks of dormancy were compared to ones from newly hatched flies and flies kept at 25°C and 12L:12D for 1 or 8 weeks after immunolabeling with antisera to HRP, discs large (*Dlg*) and the synapse protein bruchpilot (*Brp*). As shown previously (Beramendi et al., 2007), we found that the HRP immunolevel decreased with age in the NMJ, but a novel finding is that it remained high in flies exposed to diapause conditions (Figures 4A–F; Supplementary Figures S1A,B). The HRP antiserum recognizes a neuron-specific isoform of the beta subunit of Na/K ATPase nervana (Sun and Salvaterra, 1995). An age-dependent decrease in the expression of *nervana* has been demonstrated (Zou et al., 2000). The postsynaptic portion of the NMJ is characterized by a subsynaptic reticulum (SSR), which can be labeled with antiserum to discs large (Dlg), a member of a family of membrane-associated guanylate kinase homologs associated with the SSR (Lahey et al., 1994). The intensity of Dlg immunolabeling was not affected by aging (see also Beramendi et al., 2007) or dormancy (Figures 4D,E, Supplementary Figure S1C). The synaptic boutons as measured in HRP labeled specimens increase in size with age and often merged under normal conditions (see also Beramendi et al., 2007; Wagner et al., 2015), but not under diapause conditions (Figure 4G). The number and length of axon branches of the NMJ were not affected by aging or diapause (Figures 4H,I), although we discovered that the branch number was higher in flies sampled just after eclosion, and that this number remained in flies taken to diapause conditions after eclosion (Figure 4H). Finally, antiserum to Brp produced presynaptic punctate labeling that did not change significantly in flies kept in control, aging and diapause conditions (Supplementary Figures S2A–C). Thus, in summary, diapause treatment ameliorated the normal aging related changes in NMJ morphology such that they resembled those of newly hatched flies.

### Pigment-Dispersing Factor Producing Neurons and Other Correlates with Activity Rhythms and Sleep after Aging and Dormancy

**Figure 5:**
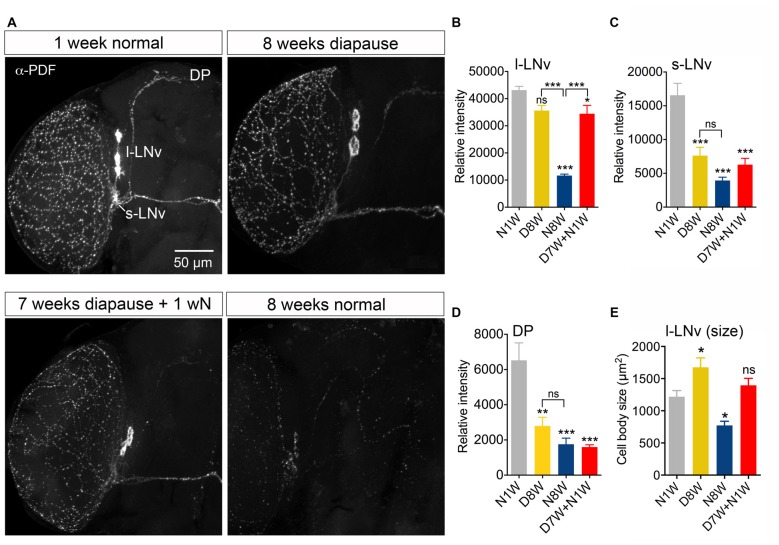
**Pigment-dispersing factor (PDF) immunolabeling of clock neurons decreases with aging, but less so during dormancy**. Antiserum to PDF (α-PDF) labels small (s-LNv) and large (l-LNv) lateral ventral clock neurons in the fly brain. All specimens were sampled for fixation and subsequent immunolabeling 2 h after onset of light. **(A)** PDF immunolabeling diminishes after 8 weeks under normal conditions, but not after 8 weeks under diapause conditions. One week of recovery from 7 weeks dormancy (D7W + N1W) is not sufficient to alter PDF immunolabeling (compared to D8W). DP, dorsal processes of s-LNv neurons. **(B)** Quantification of PDF immunolabeling in l-LNv cell bodies. **(C)** Quantification in s-LNv cell bodies. **(D)** Quantification in dorsal processes of s-LNv neurons. **(E)** The size of the cell bodies of l-LNv neurons decreases after 8 weeks in non-diapause conditions and increases somewhat after the same time in dormancy. Data are presented as means ± SEM, *n* = 6–8 flies from three replicates (*p < 0.05, **p < 0.01, ***p < 0.001, compared to N1W flies, as assessed by one way ANOVA followed by Tukey’s multiple comparisons).

In *Drosophila* circadian locomotor rhythms are generated by a set of about 150 clock neurons in the brain (see Nitabach and Taghert, 2008). Two subsets of clock neurons utilize PDF as an output, the l-LNv and s-LNv cells, and interference with PDF signaling produces major disturbances in behavioral rhythms (Nitabach and Taghert, 2008). An earlier study showed that PDF immunolabeling decreases in clock neurons of aging flies, the daily activity was reduced, and during constant darkness the free-running period was increased and its power diminished (Umezaki et al., 2012). Our data show that 8-week-old flies displayed strongly reduced PDF immunolabeling in clock neurons compared to 1-week-old controls (Figures 5A–C). During dormancy the PDF level in the l-LNv cells did not drop significantly (Figures 5A,B), whereas in the s-LNv cells the immunolabeling was significantly reduced (Figures 5A,C), also in their axon terminations (Figure 5D). After 1 week of recovery from diapause conditions the PDF level remained the same as after 8 weeks of dormancy, except in the s-LNv axon terminations (Figures 5A–D). The size of the l-LNv cell bodies increased in flies kept for 8 weeks in dormancy, and diminished in flies kept for the same time under non-diapause conditions (Figure 5E). The s-LNv cells are too small to obtain reliable measurements. The findings on PDF levels and cell size are summarized in Tables 1, 2.

**Table 1:** **Effects of dormancy and aging on immunofluorescence of neuronal markers**.

Neuron/neuropil	Marker	8W diapause^1^ (D8W)	8W normal^1^ (N8W)	D8W - N8W^2^	Figure
NMJ	HRP	nc	↓***	–	Figure 4
l-LNv (clock cells)	PDF	nc	↓***	–	Figure 5
s-LNv (clock cells)	PDF	↓***	↓***	nc	Figure 5
PAL (dopaminergic)	TH	nc	↓***	–	Figure 6
PPM1/2	TH	nc	↓***	–	Figure 6
PPM3	TH	↓***	↓***	↓***	Figure 6
PPL1	TH	nc	↓***	–	Figure 6
PPL2	TH	↓*	↓***	↓***	Figure 6
Brain neurons	5-HT	nc	↓*	–	Supplementary Figure S4
Brain DLP	Crz	↓***	↓**	–	Figure 7
Brain IPCs	DILP2	↓***	↓***	↓*	Figure 7
Brain Ipc-1	ITP	↓***	nc	–	Figure 8
Posterior neurons	FMRFa	↓***	↓***	nc	Supplementary Figure S5
Fanshaped body U	TK	↓***	↓***	nc	Supplementary Figure S6
Fanshaped body L	TK	nc	↓***	–	Supplementary Figure S6
Antennal lobe	TK	↓***	↓***	nc	Supplementary Figure S6
Mushroom body	sNPF	nc	↓***	–	Supplementary Figure S7
Fanshaped body	sNPF	nc	↓***	–	Supplementary Figure S7

Notes: ^1^Compared to 1 week old normal (N1W) conditions. ↓, decreased level, nc, no significant change. ^2^Comparison between D8W and N8W where both are significantly different from N1W. *p < 0.05, **p < 0.01, ***p < 0.001, as assessed by one-way analysis of variance (ANOVA).

**Table 2:** **Effects of dormancy and aging on cell body or neuropil size**.

Neuron/neuropil	Marker	8w diapause^1^	8w normal^1^	Figure
l-LNv	PDF	↑*	↓*	Figure 5
PPL1	TH	nc	↓**	Supplementary Figure S8
Brain neurons	5-HT	↑***	↓***	Supplementary Figure S4
Brain Ipc-1	ITP	nc	↓***	Figure 8
Mushroom body P^2^	sNPF	↑*	nc	Supplementary Figure S7
Brain neurons S^3^	FMRFa	↑***	↑*	Supplementary Figure S5
Brain neurons L^4^	FMRFa	nc	nc	Supplementary Figure S5

^1^↑ increased size; ↓, decreased size; nc, no change. ^2^Diameter of mushroom body peduncle. ^3^S, small neurons. ^4^L, large neurons. *p < 0.05, **p < 0.01, ***p < 0.001, as assessed by one-way ANOVA.

### Dopaminergic and Serotonergic Neurons Are Differentially Affected by Aging and Dormancy

Dopaminergic neurons in *Drosophila* have long served in models of Parkinson’s decease, oxidative stress and aging (see White et al., 2010; Muñoz-Soriano and Paricio, 2011). Antiserum to the rate limiting enzyme in dopamine biosynthesis, TH, is a good marker for dopamine expression in the fly CNS (Nässel and Elekes, 1992; Friggi-Grelin et al., 2003) and has been utilized for analysis of morphology and labeling intensity of dopaminergic neurons exposed to aging and genetic manipulations (see Neckameyer et al., 2000; White et al., 2010; Shih et al., 2015).

**Figure 6:**
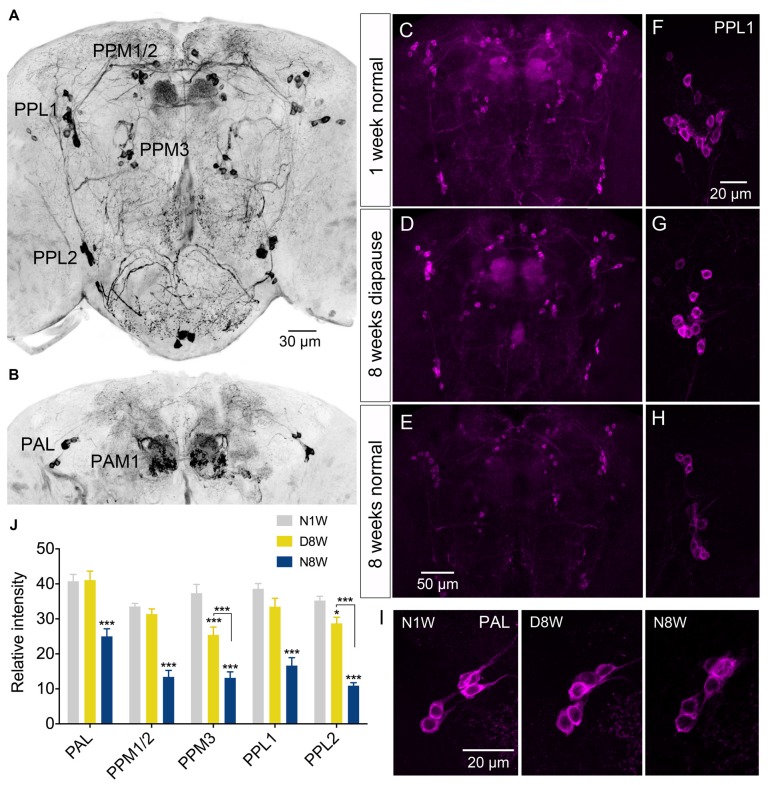
**The tyrosine hydroxylase (TH) immunoreactivity in neurons of the brain decreases with normal aging, but less so during dormancy. (A,B)** TH immunolabeling reveals several groups of neurons in the brain, six of which are indicated here by acronyms (**A** shows more anterior and **B** more posterior cell bodies). **(C–E)** Overviews of brains with TH immunolabeled neurons, representing the three different experimental groups. **(F–H)** Details of cell bodies of PPL1 neurons. **(I)** Details of cell bodies of PAL neurons. **(J)** Quantification of TH immunolabeling in cell bodies of five neuron groups. Note that in flies aging for 8 weeks under normal conditions (N8W) the TH immunolabeling is drastically decreased in all neuron types. Only in two cell types (PPM3 and PPL2) there is a slight decrease of TH immunolabeling in dormant flies. Data are presented as means ± SEM, *n* = 7–8 flies from three replicates (*p < 0.05, ***p < 0.001, compared to N1W flies, as assessed by two-way ANOVA followed by Tukey’s multiple comparisons).

Thus, we examined TH immunolabeling in flies kept for 8 weeks under diapause conditions and for the same timespan at 25°C and 12L:12D, compared to 1 week old control flies. Five groups of dopaminergic neurons were analyzed (Figure 6): PAL, PPM1/2, PPM3, PPL1 and PPL2 (terminology according to Nässel and Elekes, 1992). All these neurons displayed a strong decrease in TH immunolabeling after 8 weeks at 25°C and 12L:12D, whereas in flies kept in dormancy PAL, PPM1/2 and PPL1 neurons showed the same immunolabeling as in 1 week controls, and in the others it decreased far less than in normally aging neurons (Figure 6). Hence, TH levels decrease during aging as shown earlier (Neckameyer et al., 2000; White et al., 2010; Shih et al., 2015), but were retained under diapause conditions. We also measured the size of some of these cell bodies (PPL1) and found no change after 8 weeks of diapause conditions, whereas in normally aging flies the cell bodies were significantly smaller (see Supplementary Figures S7A,C). The findings on TH levels and cell size are summarized in Tables 1, 2. It can be noted that it has been shown earlier the number of TH expressing neurons remain the same in young and old flies, suggesting that no apoptosis occurs with aging (White et al., 2010). In accordance with this, we found that the numbers of TH-immunolabeled cell bodies in the neuron groups tested above remain the same in young flies, 8 week old ones, and flies kept in dormancy for 8 weeks (Supplementary Figure S3).

Serotonergic neurons were included in our analysis since this monoamine is important in circuits controlling behaviors known to deteriorate with age, such as circadian activity and sleep (Yuan et al., 2005, 2006), as well as memory functions (Sitaraman et al., 2012). We monitored serotonin-immunofluorescence in a set of posterior neuronal cell bodies of the brain and found that flies kept for 8 weeks under non-diapausing conditions displayed decreased fluorescence compared to diapausing and 1 week control flies (Supplementary Figures S4A–D). Compared to young controls, the size of these cell bodies increased significantly during dormancy and decreased after aging under non-diapausing conditions (Supplementary Figure S4E). Thus, diapause also seems to ameliorate the normal senescence of serotonergic neurons.

### Peptidergic Neuroendocrine Cells, Dormancy and Aging

**Figure 7:**
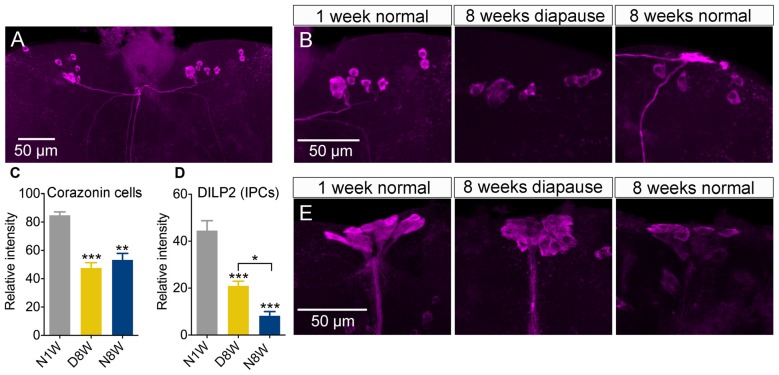
**Peptide levels in brain neuroendocrine cells are affected by aging and dormancy. (A–C)** Corazonin immunoreactive neurons display an equal decrease of labeling after 8 weeks of normal aging and 8 weeks of diapause. **(D,E)** Insulin producing cells (IPCs) can be labeled with antiserum to DILP2. The DILP2 immunolabeling decreases under both conditions, but significantly more after normal aging (non-diapause conditions). Data are presented as means ± SEM, *n* = 6–8 flies (corazonin) *n* = 6–10 flies (DILP2), each from three replicates (*p < 0.05, **p < 0.01, ***p < 0.001, compared to N1W flies, as assessed by one-way ANOVA followed by Tukey’s multiple comparisons).

Apart from the PDF neurons, we also investigated peptide expression in a few other sets of brain neuroendocrine cells exposed to our experimental conditions. The first set produces corazonin, a peptide known to participate in stress signaling (Zhao et al., 2010; Kubrak et al., 2016). The corazonin immunolabeling decreased after 8 weeks both in diapause and control conditions compared to 1 week old flies (Figures 7A–C).

Next we measured DILP2 in insulin producing median neurosecretory cells since insulin signaling appears to play major roles in diapause, stress responses, lifespan and aging (Broughton et al., 2005; Kubrak et al., 2014; Kučerová et al., 2016). We found the strongest reduction of immunolabeling in flies kept under non-diapause conditions, but a significant decrease also after 8 weeks of diapause (Figures 7D,E), although the decrease was significantly stronger after normal aging.

**Figure 8:**
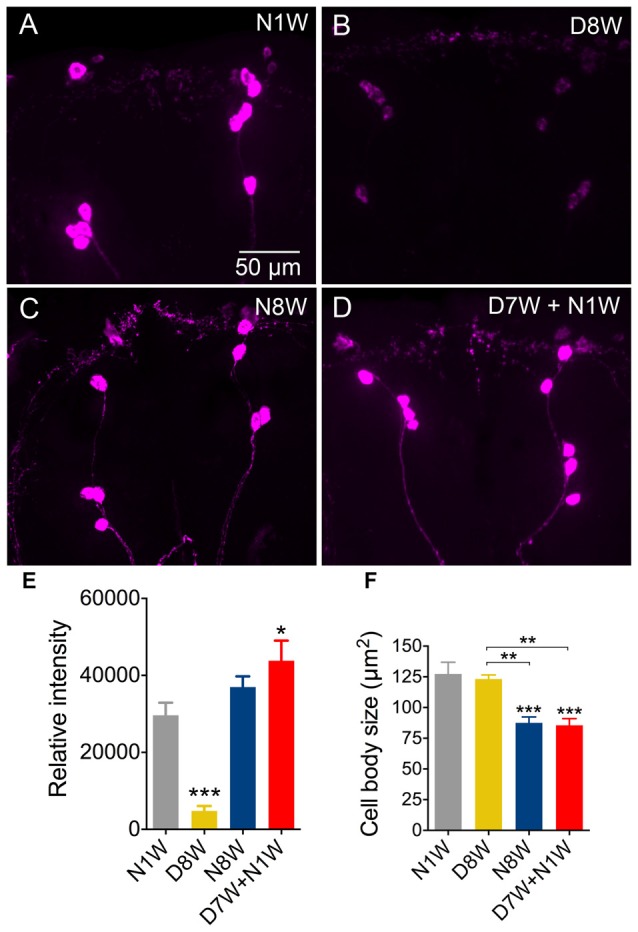
**Ion transport peptide (ITP) immunolabeling in brain neuroendocrine cells is downregulated during diapause, but recovers rapidly. (A–E)** Immunolabeling with antiserum to ITP decreases after 8 weeks of diapause conditions (D8W) compared to 1-week-old flies (N1W) and normally aging flies (N8W). However, flies kept for 7 weeks in dormancy and then allowed to recover for 1-week (D7W + N1W) display stronger ITP immunolabeling than N1W and N8W flies. **(F)** The size of the ITP neuron cell bodies diminishes in N8W and D7W + N1W flies, but not in those kept in dormancy. Data are presented as means ± SEM, *n* = 6–8 flies from three replicates (*p < 0.05, **p < 0.01, ***p < 0.001, compared to N1W flies, as assessed by one-way ANOVA followed by Tukey’s multiple comparisons).

The third set of neurons tested express ion transport peptide (ITP) and we selected a subset designated ipc-1 neurons (Dircksen et al., 2008) that play a role in stress responses (Kahsai et al., 2010a). Here we noted an expression profile different from all the neurons discussed above: after 8 weeks of dormancy the ITP-immunolabeling was drastically weaker than in young controls and flies kept for 8 weeks in normal conditions (Figures 8A–E). Interestingly, 1 week of recovery from 7 weeks of dormancy was sufficient to bring up the immunolabeling to levels comparable to the 1-week-old controls (Figures 8D,E). Since the ipc-1 neurons are distinct we measured the size of their cell bodies and found a decrease with aging only under normal conditions (Figure 8F). After a week of recovery from dormancy these cell bodies displayed the same size as those from 8 weeks of normal aging (Figure 8F).

Finally, we monitored a small set of neurons in the brain reacting with antiserum to FMRFamide, but likely to produce sulfakinin peptides (DSKs), known to regulate satiety in *Drosophila* (Söderberg et al., 2012). These are bilateral groups of two small and two large neurons posteriorly in the posterior brain (Supplementary Figures S5A–C). We found that in both dormant and normally aging flies the immunofluorescence decreased in both cell groups (Supplementary Figures S5D,E) and there was a slight increase in the size of the small, but not the large cell bodies after keeping the flies in diapausing conditions for 8 weeks (Supplementary Figures S5F,G). In no case did we detect a change in the number of neuronal cell bodies with age or dormancy for the above types of neuropeptides. The findings on peptide levels and cell size are summarized in Tables 1, 2.

### Neurons of Mushroom Bodies and Central Complex in Aging and Dormancy

Since the neuronal circuits of the mushroom bodies and central complex are known to regulate behaviors that are affected by aging, such as learning and memory, locomotor activity and walking (Martin et al., 1998, 1999; Grotewiel et al., 2005; Yamazaki et al., 2014; Ismail et al., 2015), we used antibody markers to study effects of dormancy and aging on these brain centers.

The peptide *Drosophila* tachykinin (DTK) is expressed in neurons innervating the central complex and interference with DTK expression in these neurons affects walking behavior (Kahsai et al., 2010b). We monitored DTK immunolabeling in neuronal processes in the fan-shaped body and found a decrease in intensity in the upper division both in dormant and control flies after 8 weeks compared to 1-week-old flies, but the decrease was significantly less in flies kept in dormancy (Supplementary Figures S6A,C). In the lower division there was only a reduced immunolabeling after 8 weeks of dormancy (Supplementary Figures S6A,D). We also monitored DTK immunolabeling in processes of the antennal lobe and found a decrease both in 8-week-old flies kept under both conditions, albeit more pronounced in normally aging flies (Supplementary Figures S6B,E).

Each of the mushroom bodies is composed of about 2500 small neurons called Kenyon cells (Heisenberg, 2003). These have very thin axons in the mushroom body lobes, but antiserum to sNPF labels a major subpopulation (Johard et al., 2008), so we used this to obtain a measure of peptide expression in the lobes. The sNPF labeling decreased after 8 weeks under control conditions, but remained at initial levels after dormancy (Supplementary Figures S7A–D). We also used anti-sNPF as a marker for effects on the diameter of the mushroom body peduncle and found that after 8 weeks of diapause conditions there was a significant increase, but no effect of normal aging (Supplementary Figures S7E–H). This increase in diameter could be caused by a larger number of Kenyon cell axons or increased diameters of these axons. An earlier study using electron microscopy has shown that the number of Kenyon cell axons in the peduncle decrease with age (Technau, 1984). The sNPF marker is too crude to obtain a measure of axon number in the peduncle. The findings on peptide levels and neuropil size are summarized in Tables 1, 2.

### Dormancy Does Not Affect Sensitivity to Oxidative Stress in Dopaminergic Neurons

**Figure 9:**
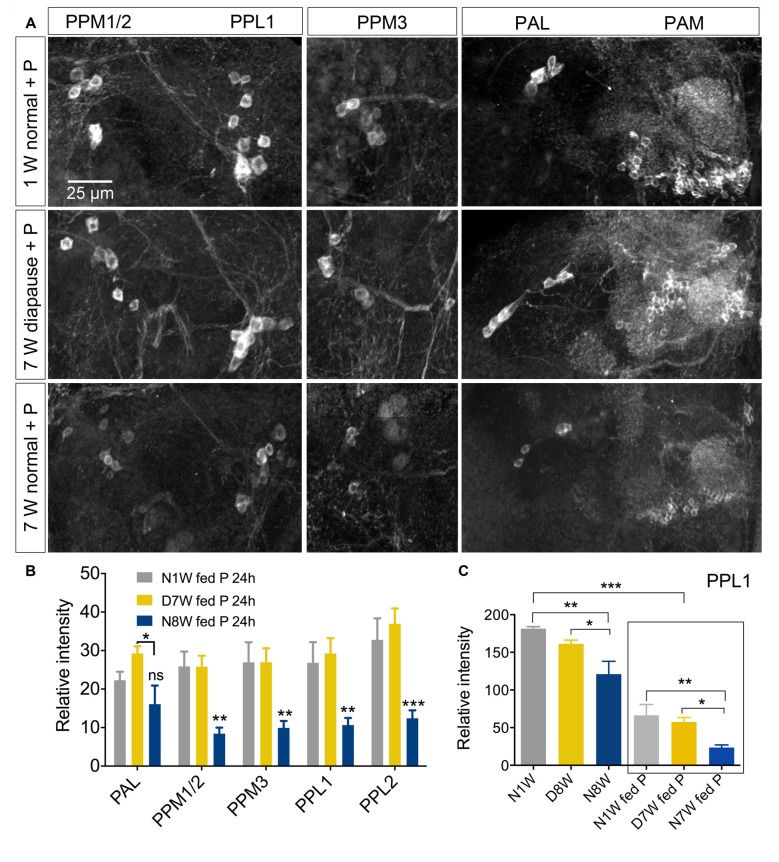
**Sensitivity of dopaminergic neurons to oxidative stress is not altered by diapause conditions. (A,B)** TH immunoreactive neurons respond to 20 mM paraquat (P) feeding by diminished immunolabeling. Here we show that the effect of paraquat feeding (fed P) for 24 h is masked by the response to normal aging. In **(B)** all data from dormant (D7W) and normally aging (N8W) are compared to 1-week-old flies (N1W). **(C)** However if we compare flies of the same aging conditions with and without paraquat feeding it is clear that paraquat reduces TH-immunolabeling in PPL1 neurons of young normal (N1W fed P), diapausing (D7W fed P) and normally aging (N7W fed P) flies. In **(C)** we compare N1W flies to D8W and N8W, N1W fed P flies to D8W fed P and N8W fed P, as well as N1W flies kept on normal food to each of those fed paraquat (in box). Data are presented as means ± SEM, *n* = 5–8 flies for anti-TH, from three replicates), sNPF (*p < 0.05, **p < 0.01, ***p < 0.001, compared to N1W flies, as assessed by two-way ANOVA followed by Tukey’s multiple comparisons).

Dopaminergic neurons are known to be very sensitive to oxidative stress both in mammals and *Drosophila* (White et al., 2010; Surmeier et al., 2011; Navarro et al., 2014). We chose to test the response of dopaminergic neurons to oxidative stress in aging flies and flies kept under diapause conditions, since it is known that diapause increases stress resistance (MacRae, 2010; Flatt et al., 2013; Kučerová et al., 2016). We exposed flies to 20 mM paraquat in the food medium for 24 h after they had been kept for 1 or 8 weeks at normal conditions or 8 weeks of diapausing conditions. Paraquat feeding reduced TH immunolabeling in flies kept under all conditions (Figure 9, Supplementary Figure S8). The 8-week-old non-dormant flies displayed the most strongly reduced TH immunolabeling both in controls and after paraquat feeding (Figure 9C). Thus, we do not see any difference in resistance to oxidative stress between normally aging and dormant flies. We also monitored the size of cell bodies of TH-immunolabeled neurons (PPL1) exposed to paraquat, aging and dormancy. The paraquat fed flies displayed smaller PPL1 cell bodies for all conditions and there was an additional decrease of cell body size in flies aging under control conditions (Supplementary Figure S8).

#### qPCR of Gene Expression in Dormant and Aging Flies

It was shown previously that keeping flies under diapause conditions alters numerous gene transcripts compared to control flies (Kubrak et al., 2014; Kučerová et al., 2016). These earlier studies were performed on whole animals and did not analyze transcript changes in flies aging under non-diapause conditions. Here we performed qPCR on head extracts of young, aging and dormant flies to probe changes in 11 genes expressed in the brain. These are genes encoding the insulin-like peptides *dilp1, dilp2, dilp3, dilp5 and dilp6*, the insulin receptor, *InR*, the subunit of PI3K, *dp110*, the translational inhibitor *Thor*, also known as eukaryotic initiation factor 4 binding protein (*4EBP*), the transcription factor Dimmed (*Dimm*), as well as the biosynthetic enzymes *Th* and choline acetyltransferase (*ChAT*). The first seven of these genes relate to IIS, *Dimm* is known to be expressed in adult peptidergic neurons and may play a role in maintenance of their secretory capacity over lifespan (Hewes et al., 2006), and finally, *Th* and *ChAT* levels may provide information about dopamine and acetylcholine signaling.

Of the 11 genes analyzed only six genes displayed small, but significant, changes in RNA levels after dormancy or aging (Supplementary Figure S9). Transcripts of the insulin-like peptides, *dilp1, dilp2, dilp3* and* dilp5*, were found to be upregulated in dormant flies only, whereas *dilp6* was not significantly altered (Supplementary Figure S9). Of these transcripts *dilp3* was downregulated in normally aging flies (N8W) and *dilp5* slightly upregulated also in N8W flies. The *InR* and *dp110* transcripts displayed no significant changes, whereas *4EBP* increased in dormant flies only. Finally, ChAT was upregulated in dormant flies, but *Th* and *Dimm* transcripts did not change. For the genes related to IIS the changes in transcript levels in dormant flies compared to young flies were similar to earlier findings, except for *dilp6* and *dp110* (Kubrak et al., 2014; Kučerová et al., 2016). However, these previous investigations monitored transcripts in whole flies, and here we used heads only. It is noteworthy that the *Th* transcript levels did not change in flies kept for 8 weeks in dormancy or normal conditions, whereas at the protein level they decreased in normally aging flies (Figure 6). A novel finding here is that transcripts in heads of flies aging for 8 weeks under non-diapausing conditions are not significantly different from young flies, except for those of *dilp3* and *dilp5*.

## Discussion

We show here that adult reproductive dormancy diminishes behavioral senescence and age-related changes of neuronal properties in *Drosophila*. The extensive increase in longevity of dormant flies was shown previously to be associated with transcriptional changes in gene sets known for their roles in aging and lifespan extension (Kučerová et al., 2016). Furthermore, signaling pathways critical for increased stress tolerance and improved immunity have been strongly implicated in the diapause syndrome of different insects (MacRae, 2010; Sim and Denlinger, 2013; Kučerová et al., 2016). A previous study suggests that extended lifespan during dormancy is also associated with reduced somatic senescence, as seen for example in the intestinal epithelium (Kubrak et al., 2014), and we can now extend this to the central nervous system and NMJ.

Negative geotaxis is commonly used to test age-related decline in reflex locomotor behavior in *Drosophila* (Gargano et al., 2005; Bhandari et al., 2007). It was previously shown that negative geotaxis senescence in long-lived flies was countered by reducing systemic IIS by various genetic interventions (Martin and Grotewiel, 2006; Ismail et al., 2015). We find here that this behavior does not deteriorate noticeably over 7 weeks of dormancy, in contrast to flies aging for the same duration under non-diapause conditions. This dramatic delay in the onset of negative geotaxis senescence is thus compatible with the observed reduction in systemic IIS during diapause (Kučerová et al., 2016). Since negative geotaxis is considered to be partly dependent on the peripheral nervous system and muscles, and therefore influenced by systemic IIS (Martin and Grotewiel, 2006; Demontis and Perrimon, 2010; Ismail et al., 2015), we analyzed NMJ structures in our experimental flies. We observed that the axon terminations of motor neurons on body wall muscles display specific morphological changes during aging under control conditions (see also Beramendi et al., 2007; Wagner et al., 2015), but not after the same time in dormancy. Thus, the amelioration of age-related changes in the NMJ under diapause conditions likely contributes to the delay in the onset of senescence of negative geotaxis behavior. However, some aspects of this behavior may additionally be dependent on neuronal circuits within the CNS that regulate activity, such as for instance circuits using the neuropeptide PDF (Toma et al., 2002; Mertens et al., 2005) or dopamine (White et al., 2010; Riemensperger et al., 2013).

Daily locomotor activity rhythms are controlled by a complex set of clock neurons in the brain (Helfrich-Förster et al., 2007; Nitabach and Taghert, 2008) and sleep is mainly regulated by neurons dependent on homeostatic mechanisms (Shaw et al., 2000; Mattis and Sehgal, 2016). Several neuronal circuits, including the central complex and mushroom bodies, have been implicated in sleep-wake behavior (Griffith, 2013) and these utilize neurotransmitters such as dopamine, octopamine and GABA, but also neuropeptides like AKH, DILPs and leucokinin seem to play roles in sleep regulation (Robertson and Keene, 2013; Metaxakis et al., 2014). It is known in both mammals and *Drosophila* that circadian rhythms and sleep deteriorate with increasing age (Shaw et al., 2000; Koh et al., 2006; Froy, 2011; Umezaki et al., 2012). Earlier studies also showed that the main effect of aging was a reduction in daytime activity (Umezaki et al., 2012). We found here that activity and sleep patterns similarly declined during normal aging in *Drosophila*, and we show that sleep fragmentation occurs in both day and nighttime sleep during aging. Diapause treatment ameliorates the aging related changes in daytime sleep, but is less effective at ameliorating nighttime sleep fragmentation. During the dark period diapause treated flies are more active and sleep less than the old and young flies kept in control conditions. Thus, dormancy differentially affects the mechanisms underlying day and nighttime sleep and activity.

One of the main signals in the clock circuit is the peptide PDF, produced by a small set of lateral clock neurons (s-LN_v_ and l-LN_v_; Nitabach and Taghert, 2008). It was shown earlier that PDF immunolabeling in LN_v_s diminish with increasing age, and that over expression of PDF targeted to these neurons could rescue the activity rhythm (Umezaki et al., 2012). Here we confirmed this decrease in PDF immunolabeling and furthermore showed that flies kept for 8 weeks in diapause conditions maintain high levels of the peptide. Since the age-induced alteration of sleep behavior could be countered by overexpression of PDF in LN_v_s it is suggestive that the ameliorated sleep senescence seen in flies kept in dormancy is at least partly due to maintenance of higher PDF levels. Also TH levels in dopaminergic neurons, which play a role in sleep regulation and general arousal (White et al., 2010; Griffith, 2013), were found in our study to be affected by aging, but much less so during dormancy.

Senescence of locomotor activity and walking behavior has been described in *Drosophila* (Martin and Grotewiel, 2006; Serway et al., 2009; Jones and Grotewiel, 2011; Ismail et al., 2015). Walking behavior is controlled by circuits in the brain such as the central complex (Strauss, 2002; Kahsai et al., 2010b), and general locomotor activity also depends on mushroom bodies and dopaminergic circuits (Martin et al., 1998; Serway et al., 2009; White et al., 2010). Similarly to the effect on startle induced negative geotaxis senescence, dormancy also delayed the onset of spontaneous exploratory walking senescence. Correlated with this we found that levels of TH in most dopaminergic brain neurons, including PPM3 neurons that innervate the fan shaped body of the central complex (White et al., 2010), did not diminish in flies kept in dormancy, but did so in flies aging under normal conditions. Also sNPF in mushroom body lobes, as well as sNPF and tachykinin in the fan-shaped body displayed the same changes with normal aging, but did not diminish as much in flies kept in dormancy. Tachykinin and sNPF are only two out of many neuropeptides in neurons of the central complex (see Kahsai and Winther, 2011), and it may be that others also maintain higher levels in dormant flies since transcripts of other peptides known to be expressed in this neuropil (e.g., neuropeptide F and allatostatin A; Kahsai and Winther, 2011) were found to be upregulated under diapause conditions (Kučerová et al., 2016).

It was shown earlier that targeted overexpression of TH in specific dopaminergic neurons that normally display an age-related decline in dopamine levels, alleviated diminishment of male courtship behavior in aging *Drosophila* (Kuo et al., 2015). Another study demonstrated that elevating TH levels in dopaminergic neurons restored an aging-associated decline in cold sensitivity in a temperature preference behavior (Shih et al., 2015). These two studies, as well as the one on sleep and PDF (Umezaki et al., 2012), suggest that behavioral senescence can be diminished by restoring levels of neuromodulators in appropriate neurons. Thus, we speculate that the negligible senescence of locomotor behavior in flies during dormancy is caused, at least partly, by relevant neurons maintaining production of neuromodulators at levels seen in younger flies.

Peptide hormones such as ITP, corazonin and DILP2 display somewhat different responses to aging and dormancy than the neuropeptides and monoamines discussed above. ITP immunolabeling diminishes in dormant flies, but not in normally aging ones. The other two peptides diminish during both diapause and non-diapause conditions. However, at the transcript level we found here that *dilp1, 2, 3* and* 5* in heads were upregulated only in flies kept in dormancy, and an earlier analysis showed upregulation of corazonin and ITP transcripts in diapausing flies together with those of 22 other neuropeptide genes (Kučerová et al., 2016). It should be noted that, except for DILPs and IIS, discussed below, it is not clear how to interpret these neuropeptide transcript levels in relation to strength of peptide signaling. We found previously that, although several *dilp* transcripts are upregulated during dormancy, transcript profiles of genes downstream of the InR suggest that systemic IIS is downregulated during dormancy (Kučerová et al., 2016). Therefore, that study concluded that hormonal DILP release is diminished during dormancy, and that the elevated *dilp* transcript levels do not reflect systemic action. In support of this, another study showed that the *dilp2* transcript level did not correlate with actual systemic release of DILP2 (Park et al., 2014). A novel finding here is that the transcript levels of dilps (except dilp3 and 5) and other genes do not change significantly during aging under normal conditions.

Adult diapause, or dormancy, is a state where cellular and systemic stress protection is increased (Tatar and Yin, 2001; MacRae, 2010; Flatt et al., 2013; Kučerová et al., 2016). Thus oxidative stress and other detrimental effects on aging of the CNS are presumably diminished. Yet we did not find evidence for increased resistance to paraquat in dopaminergic neurons of flies kept in diapause conditions. Possibly the 20 nM paraquat is highly toxic and deleterious for neurons even with increased stress resistance.

Based on studies of dopaminergic neurons and PDF expressing clock neurons, discussed above (Umezaki et al., 2012; Kuo et al., 2015; Shih et al., 2015), we suggest that the reduced senescence of the behaviors studied here is caused, at least in part, by the maintenance of neurotransmitters and neuropeptides at youthful levels in brain neurons. So what is the mechanism behind the reduced senescence of the brain neurons? Although systemic IIS, which is downregulated during diapause (Kučerová et al., 2016), is an obvious candidate to underlie the reduced aging of brain neurons, lifespan extending systemic reductions in IIS in flies have been shown to have no beneficial effect on exploratory walking senescence (Ismail et al., 2015). Ismail et al. (2015) also showed that reduced IIS in brain neurons is deleterious to age-related behavior, but that study could not separate functional deficits from aging effects of reduced IIS. Here, we tested behavioral function only after flies had recovered from diapause treatment, with a concomitant return to normal IIS levels, and so we can separate functional effects of reduced IIS from aging effects. Our data are therefore consistent with reduced IIS contributing to the slowed aging of brain neurons during diapause. We propose a model whereby systemic IIS is reduced sufficiently to contribute to slowing aging of all tissues including those that normally require IIS for function or cell survival. Neurons of the brain, which are sensitive to reduced IIS, may be protected by local increases in InR expression, which optimizes IIS in neurons for slowed aging but maintains neuronal survival. This level of IIS in the brain during diapause would not be optimal for neuronal function, but we speculate that this is not required during dormancy.

In conclusion, we can add reduced behavior senescence and neuronal aging to the adult diapause syndrome. Thus, flies in reproductive dormancy are not only living on a minimal metabolic rate with increased stress resistance and reduced somatic senescence, they also seem to keep their nervous system in a state of suspended animation. The behavior of dormant flies has not been studied in detail, but it is clear that they feed at a very reduced rate, they do not display reproductive behavior (Kubrak et al., 2014), and their locomotor activity is low with a very weak daily rhythmicity (unpublished observations). After interrupting dormancy these behaviors all return to the state of young flies in a few days (Kubrak et al., 2014), and the mortality rate of recovered flies resembles that of newly hatched ones (Tatar and Yin, 2001). Furthermore, we show here that walking behavior senescence also sets in at a normal rate after transfer to non-diapause conditions. In other words, flies that have been through dormancy, recover their physiology and thereafter a normal lifespan with normal senescence ensues. This *Drosophila* laboratory model will be very useful to further unravel mechanisms in aging of the nervous system, including IIS and other gene networks that are important in ameliorating deleterious effects of aging.

## Author Contributions

SL, SB and DRN designed the study and interpreted the data. SL and SB performed the experiments; SL and DRN with inputs from SB wrote the article; DRN supervised the study and obtained funding. All authors edited and approved the article.

## Conflict of Interest Statement

The authors declare that the research was conducted in the absence of any commercial or financial relationships that could be construed as a potential conflict of interest. The reviewer SLL and handling Editor declared their shared affiliation, and the handling Editor states that the process nevertheless met the standards of a fair and objective review.

## Footnotes

^1^http://rsb.info.nih.gov/ij/

^2^www.graphpad.com
