# Supplementary material for: Behavioral Senescence and Aging-Related Changes in Motor Neurons and Brain Neuromodulator Levels Are Ameliorated by Lifespan-Extending Reproductive Dormancy in Drosophila
Source: Front Cell Neurosci. 2017 Apr 20;11:111. doi: 10.3389/fncel.2017.00111 (PMC5408790; doi:10.3389/fncel.2017.00111)
Supplement: Supplementary file 1 [file Image_1.pdf]

Supplementary Material

Behavioral senescence and aging-related changes in motor neurons and brain neuromodulator levels are ameliorated by lifespan-extending reproductive dormancy in *Drosophila*

Sifang Liao, Susan Broughton and Dick R. Nässel*

*** Correspondence:** Dick R. Nässel dnassel@zoologi.su.se

Contents: Supplementary Figures 1-9

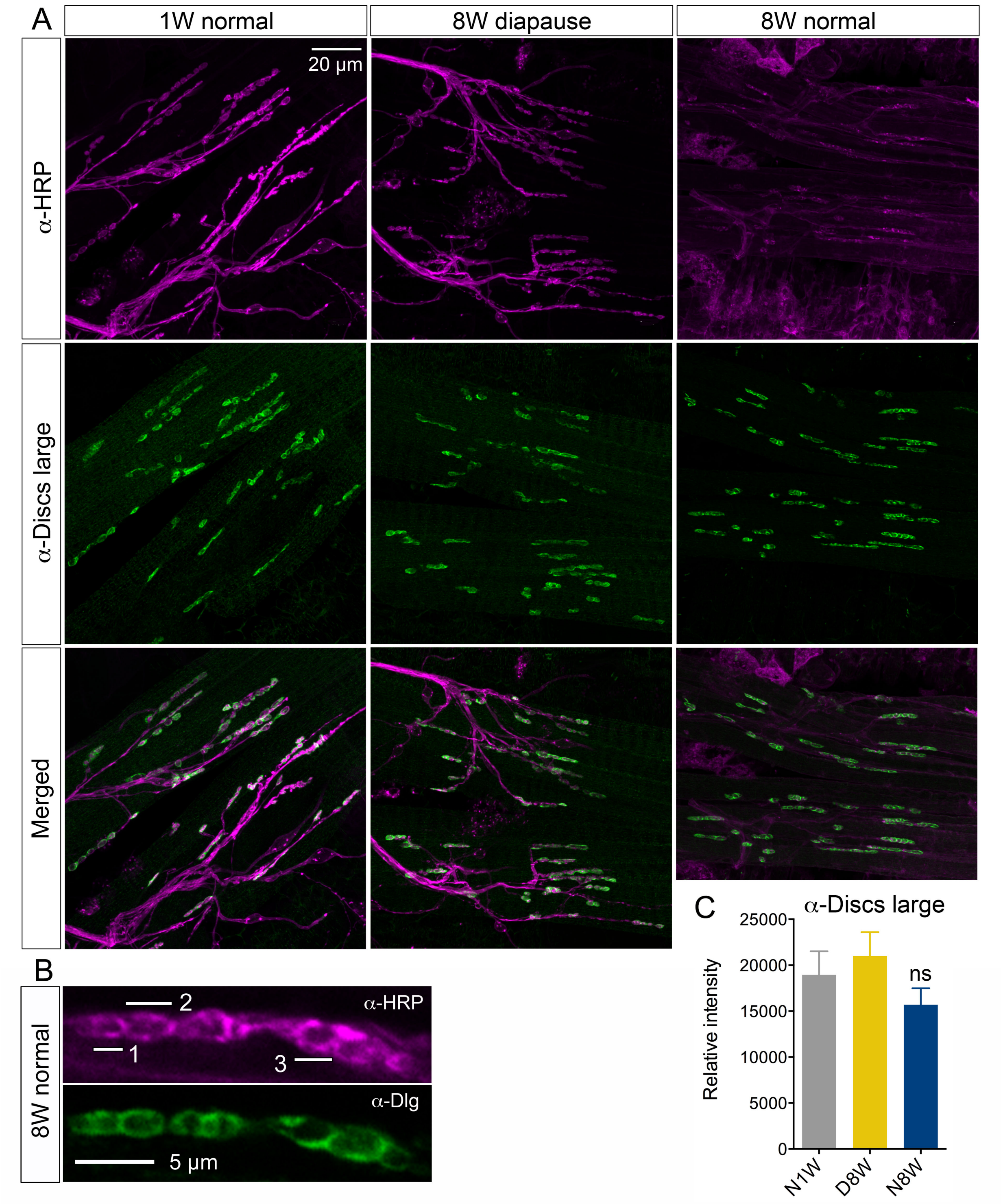

Supplementary Figure 1. Overview of NMJs analyzed in the experiments shown in Fig. 4. **A.** NMJs labeled with anti-HRP and anti-discs large in ventral abdominal muscles of flies from different conditions. The scale bar (20 µm) in upper panel refers to all panels. **B.** Diameters of anti-HRP-labeled boutons were measured as the longest diameter (as indicated by white bars 1-3). Only well delineated boutons were measured. **C.** Quantification of Discs large immunolabeling. No significant difference between flies, as assessed by one-way ANOVA with Tukey's multiple comparison (5-7 flies from 3 replicates were analyzed). This figure is accompanying Fig. 4D.

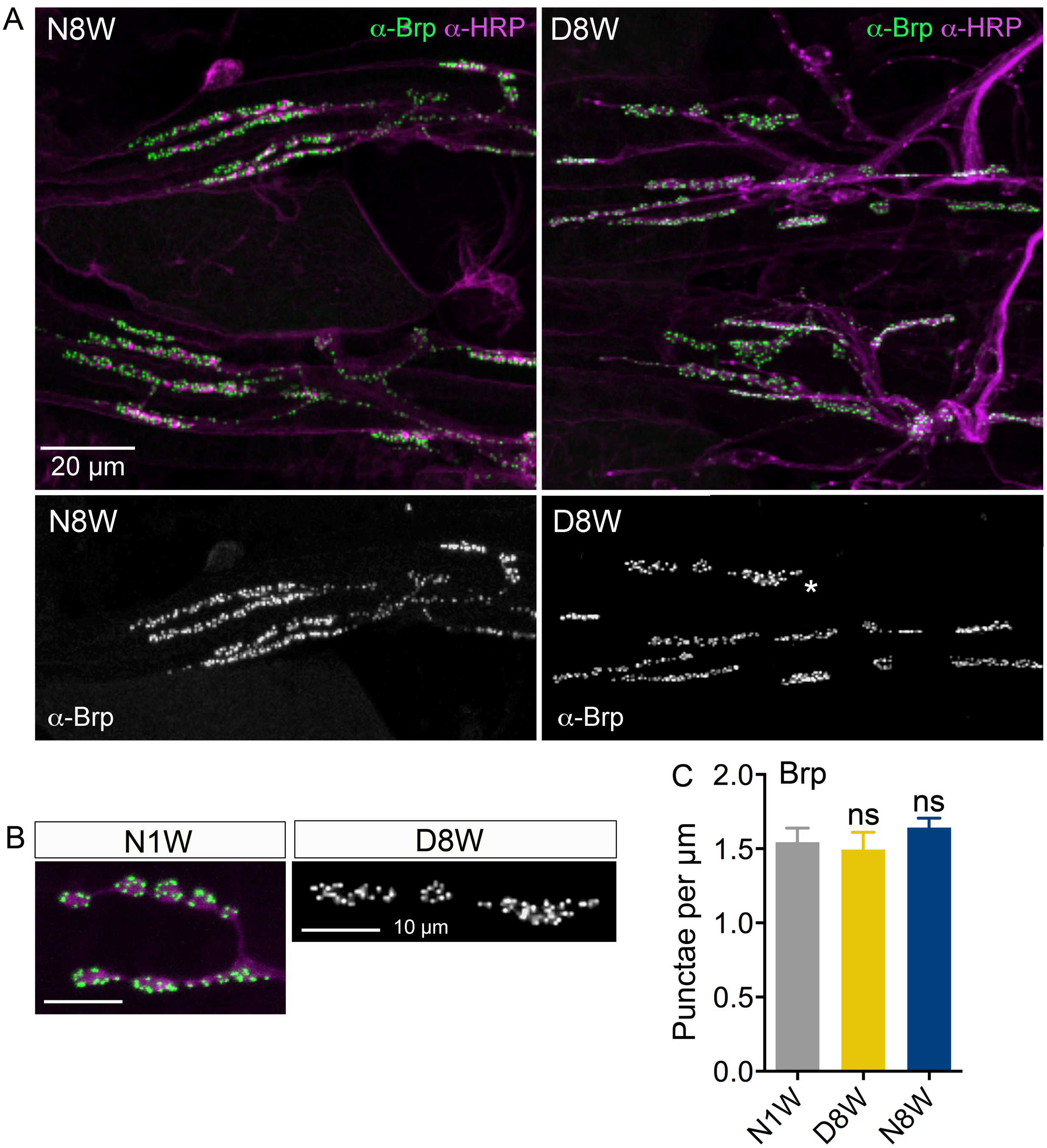

Supplementary Figure 2. The synapse protein bruchpilot distribution remains the same with aging and dormancy, whereas HRP expression diminishes with aging. **A**. Overview of NMJs labeled with antisera to HRP and bruchpilot (Brp) from flies kept for 8 weeks in diapause conditions (D8W) and normal (N8W) conditions. The Brp labeling reveals presynaptic active zones. **B.** Enlarged details of Brp labeling from N1W and D8W flies (the D8W image is from the area marked with asterisk in D8W panel in A). **C.** No difference was observed in number of Brp punctae between the three conditions. The number of Brp punctae was measured over defined segments of the axons and presented as punctae per µm. 8 branches were analysed from 8 different muscles, in each of 5-6 flies from 3 replicates. N8W and D8W flies were not significantly different from N1W flies, as assessed by one-way ANOVA with Tukey's multiple comparison. This figure is associated with Fig. 4.

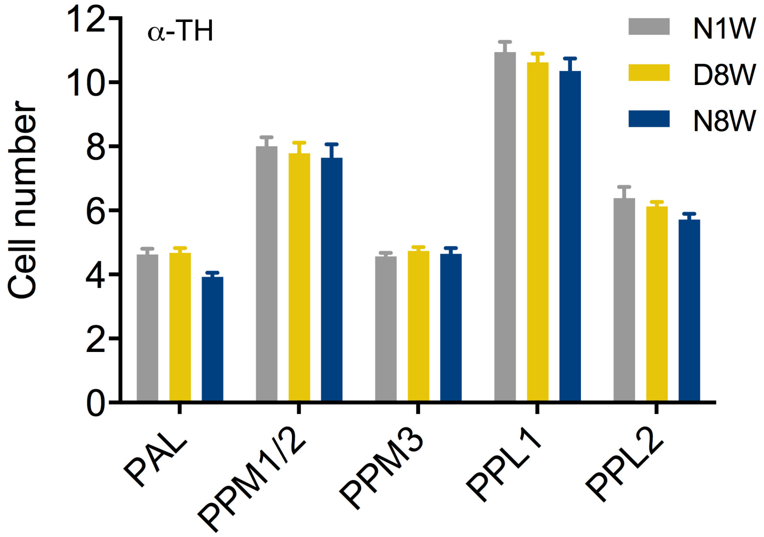

Supplementary Figure 3. The number of TH-immunolabeled neurons does not change with aging or dormancy. This graph shows the mean number of TH-positive neuronal cell bodies of each type in one brain hemisphere. There is no significant difference in neuron number between treatments. It should, however, be noted that for the N8W flies the intensity of the immunofluorescence had to be increased in Image J, since with standardized confocal settings used for imaging some cell bodies were very weakly labeled. Data are presented as means ± S.E.M, n = 7-8 flies from three replicates. For all neuron types cell body numbers in D8W and N8W flies were not significantly different from N1W flies, as assessed by two-way ANOVA with Tukey's multiple comparison. This figure is associated with Figure 6.

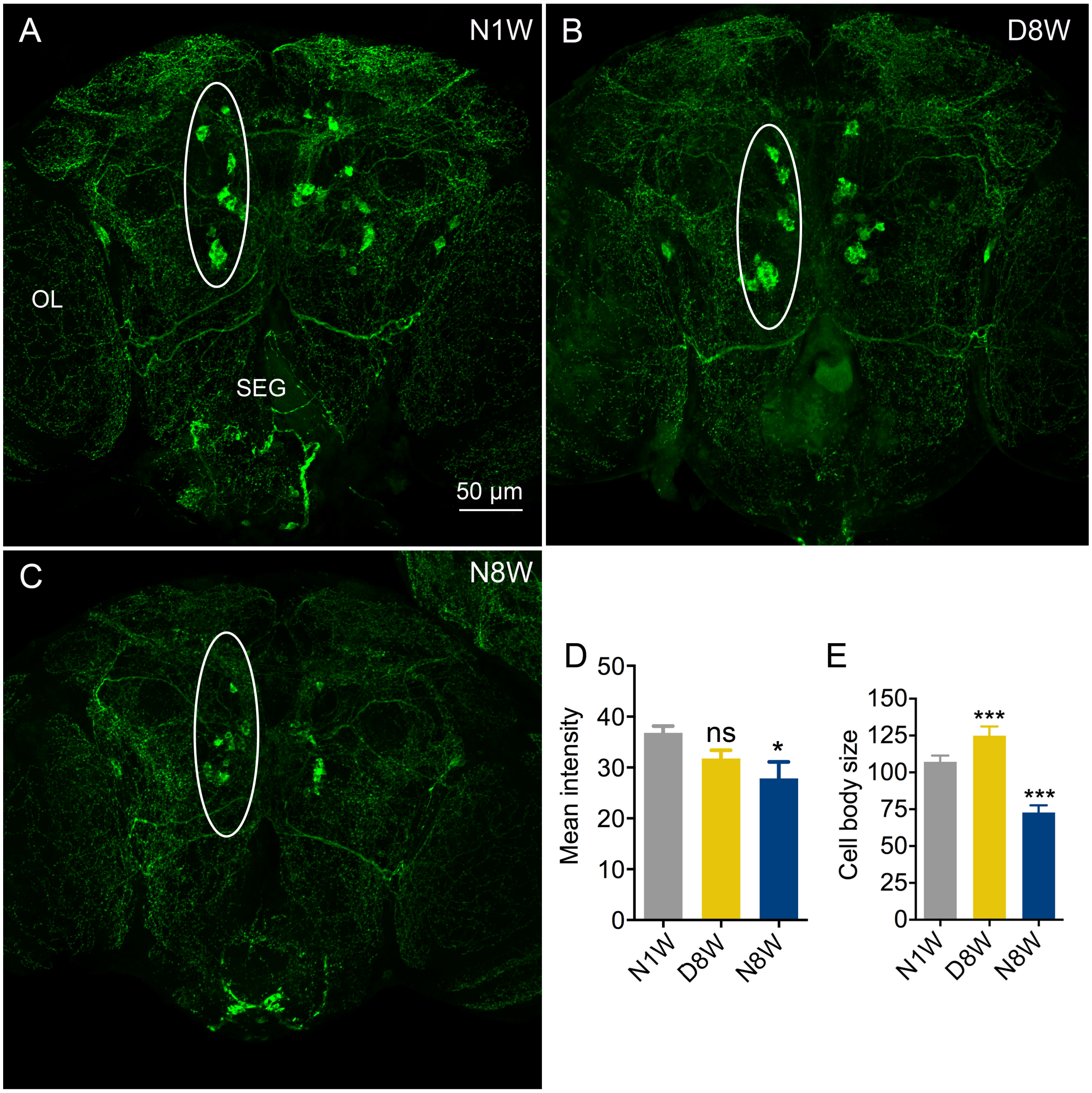

Supplementary Figure 4. Effects of aging and dormancy on serotonergic neurons in the brain include level of immunolabeling and cell body size. **A.** Flies kept for 1 week under normal conditions (N1W). Serotonin immunofluorescence was measured in the encircled cell bodies in each brain. SEG, subesophageal ganglion, OL, optic lobe. **B.** Flies kept 8 weeks in diapause conditions (D8W). **C.** Flies kept 8 weeks in normal conditions (N8W). **D.** Quantification of mean fluorescence intensity in cell bodies. **E.** Size of cell bodies in the same group. Data are presented as means ± S.E.M, n = 6–9 flies from three replicates (*p < 0.05, ***p < 0.001, compared to N1W flies, as assessed by one-way ANOVA with Tukey's multiple comparison).

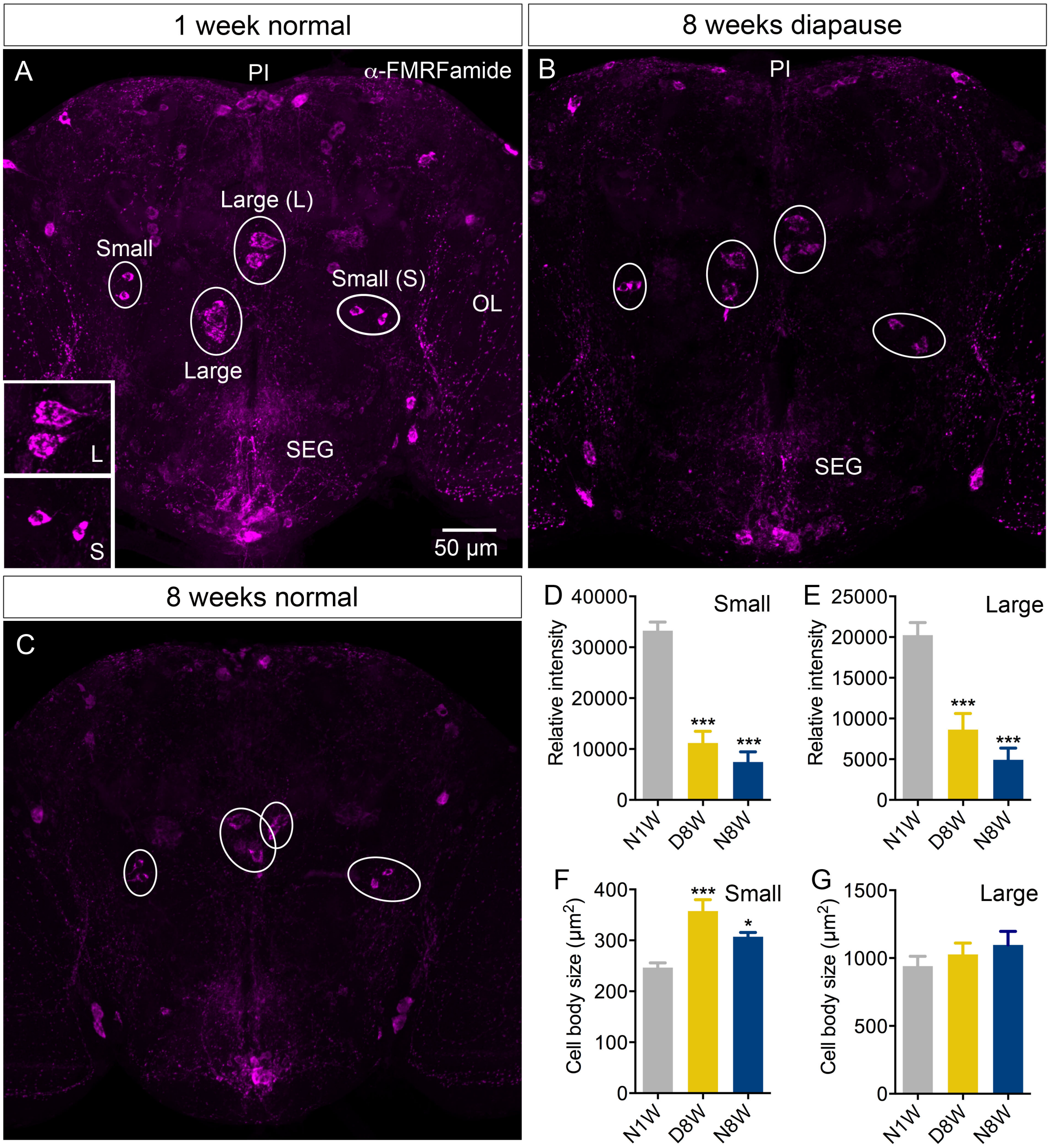

Supplementary Figure 5. Effects of aging and diapause on FMRFamide immunolabeled neurons in the brain. **A-C**. Antiserum to FMRFamide labels numerous neurons in the fly brain. Here we measured immunofluorescence and cell body size in two groups of posterior neurons, large and small ones, shown encircled in A and B. The insets L and S show details of the cells indicated large (L) and small (S) in A. PI, pars intercerebralis; SEG, subesophageal ganglion, OL, optic lobe. **D, E.** Quantification of immunolabeling in small and large neurons. The intensity decreases after 8 weeks in both diapause (D8W) and normal (N8W) conditions compared to 1 week old flies (N1W). **F, G.** The cell bodies of the small neurons increase in size in both conditions. Data are presented as means ± S.E.M, n = 7–8 flies from three replicates (*$p < 0.05$, ***$p < 0.001$, compared to N1W flies, as assessed by one-way ANOVA with Tukey's multiple comparison).

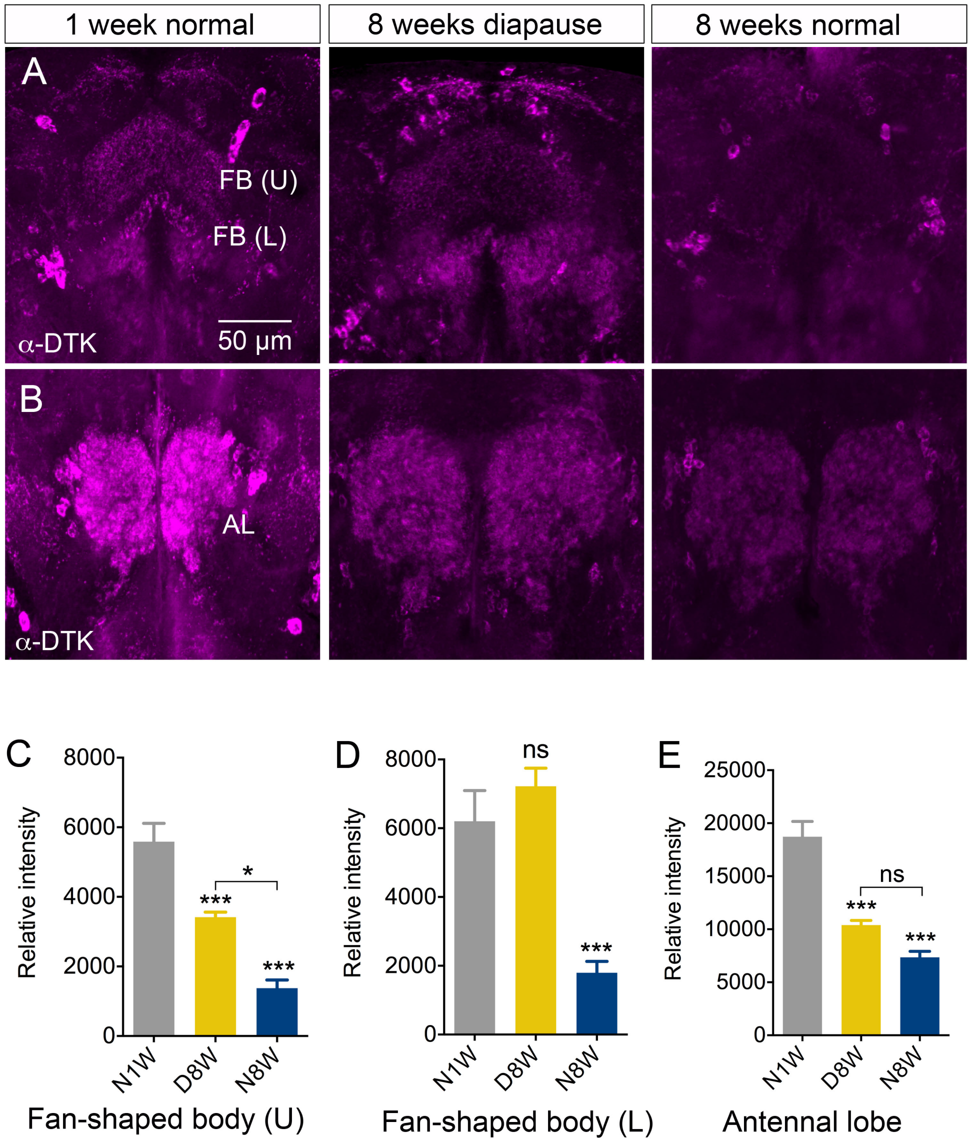

Supplementary Figure 6. Effects of aging and dormancy on tachykinin-related peptide (DTK) immunolabeled neurons in the brain. **A.** Neuron processes in the fan shaped body (FB) of the central complex were measured in upper, FB (U), and lower, FB (L), division. **B.** Arborizations of local interneurons of the antennal lobes (AL) were measured. **C-E**. Relative immunofluorescence was quantified for the three structures. A decrease was seen in both 8 W groups, except in the FB (L), where D8W flies displayed no alteration of immunolabeling. Data are presented as means ± S.E.M, n = 7–9 flies from three replicates (*p < 0.05, ***p < 0.001, compared to N1W flies, as assessed by one-way ANOVA with Tukey's multiple comparison).

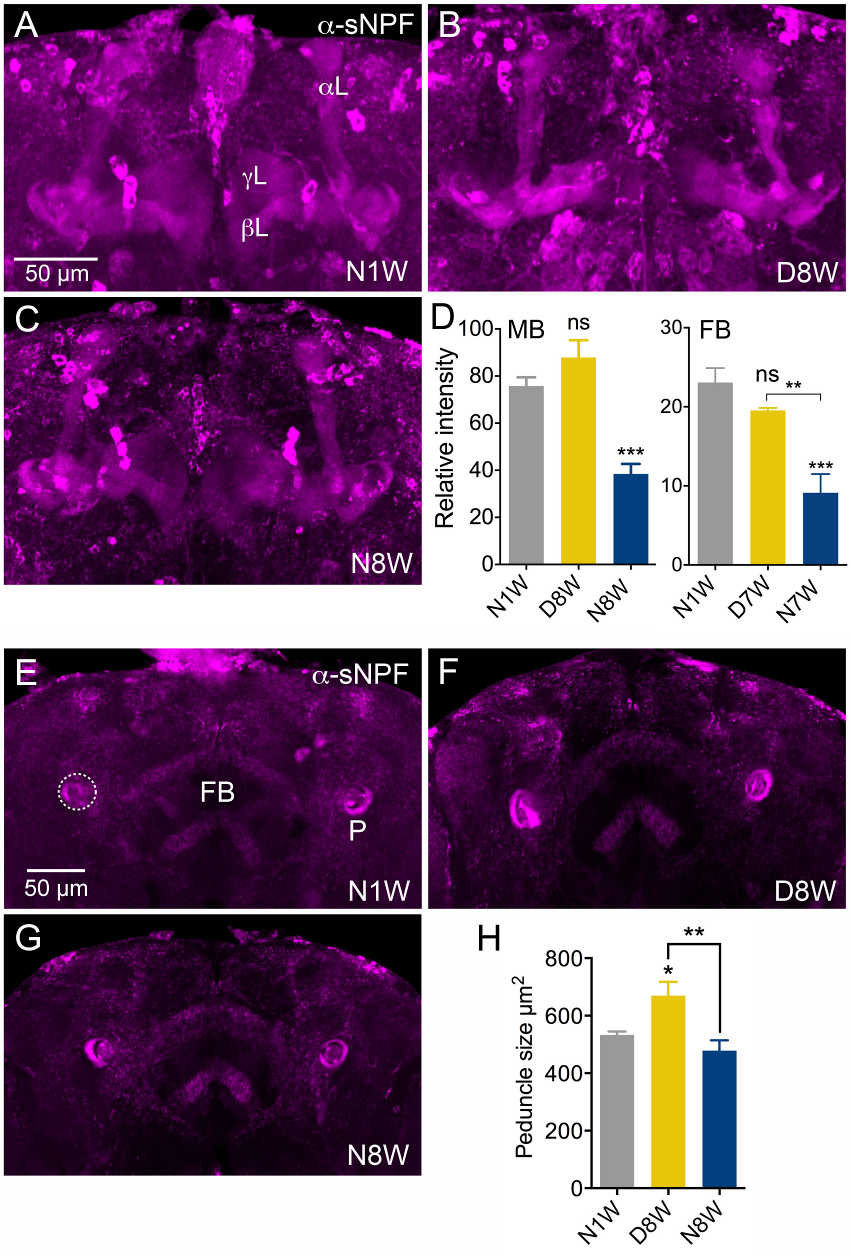

Supplementary Figure 7. Effects of aging and dormancy on short neuropeptide F (sNPF) immunolabeled neurons in the brain. **A-C.** A large portion of the intrinsic Kenyon cells of the mushroom bodies was labeled with anti-sNPF. Compared to young controls (N1W), the intensity of immunolabeling was not changed in flies kept for 8 weeks in dormancy (D8W), but significantly reduced after 8 weeks in normal conditions (N8W). **D.** Quantification of sNPF labeling in mushroom body lobes (MB) and fan-shaped body (FB). Fluorescence intensity decreased in flies aging under normal conditions. N8W is significantly lower than D8W ($p<0.001$). **E-G.** The diameter of the mushroom body peduncle (P) (seen in cross section) changed somewhat with aging and dormancy. Note that these images are shown with maximum intensity/contrast to reveal outline of the peduncles, thus fluorescence intensity in fan-shaped body (FB) is not reflecting levels shown in Suppl. Fig. 7D. **H.** Quantification of the peduncle diameter shows that it increases in diapause conditions. Data are presented as means ± S.E.M, n = 5–9 flies from three replicates (*$p < 0.05$, **$p < 0.01$, ***$p < 0.001$, compared to N1W flies, as assessed by one-way ANOVA with Tukey's multiple comparison).

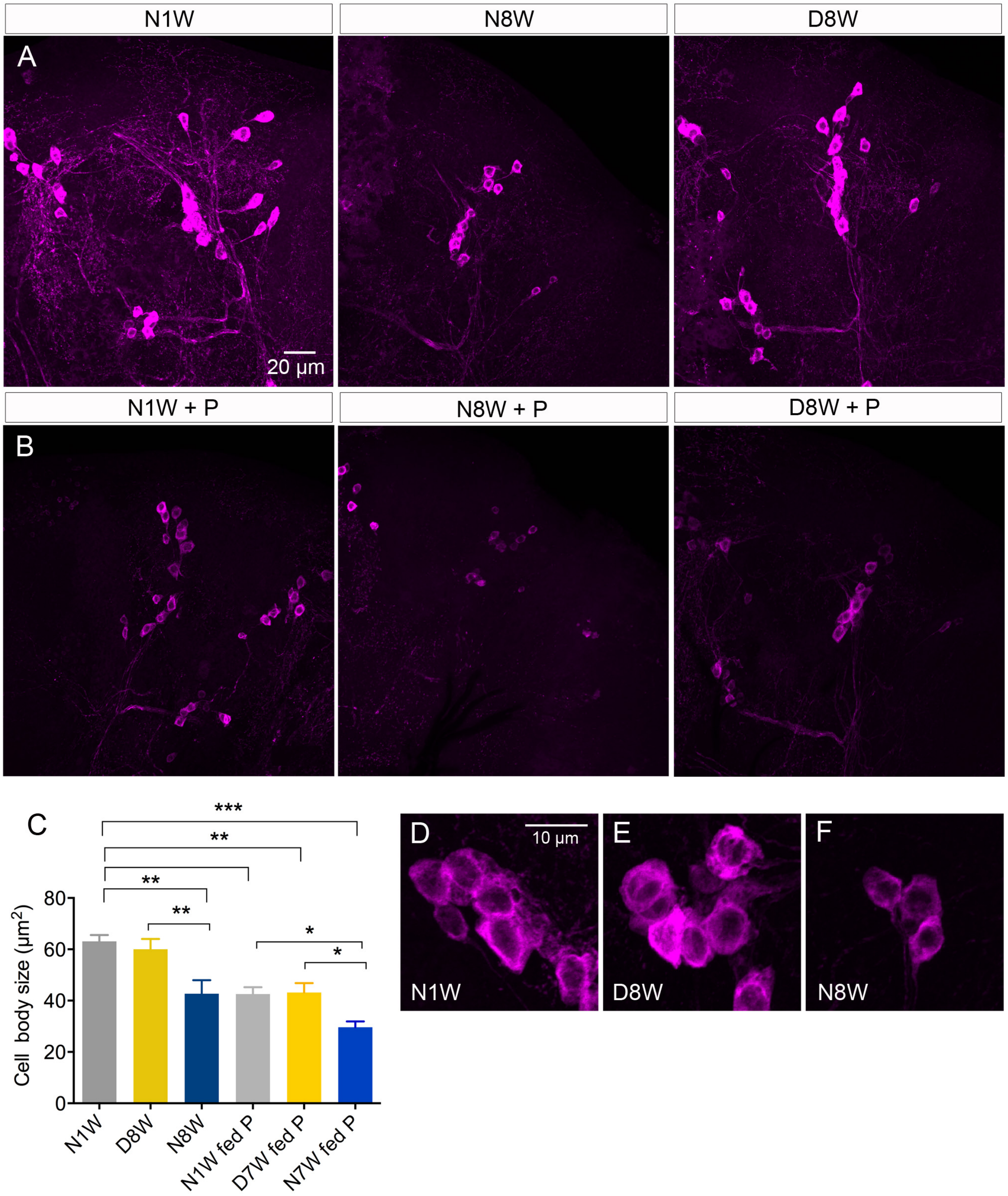

Supplementary Figure 8. Sensitivity of dopaminergic neurons to oxidative stress is not altered by diapause conditions (supplement to Fig. 9). **A, B.** Overview of dopaminergic neurons (TH-immunolabeling) in one brain hemisphere under normal (N1W, N8W) and diapause conditions (D8W) fed on normal food (**A**) or for 24 h on food with 20 mM paraquat (+P in **B**). **C-F.** The size of cell bodies of dopaminergic PPL1 neurons was affected both by aging and paraquat. Both in control food and paraquat-spiked food flies kept for 8 weeks under non-diapause conditions displayed smaller cell bodies. All groups fed paraquat display smaller cell bodies than those fed normal food only. Data are presented as means ± S.E.M, n = 7–8 flies from three replicates (*p < 0.05, **p < 0.01, ***p < 0.001, as assessed by one-way ANOVA with Tukey's multiple comparison).

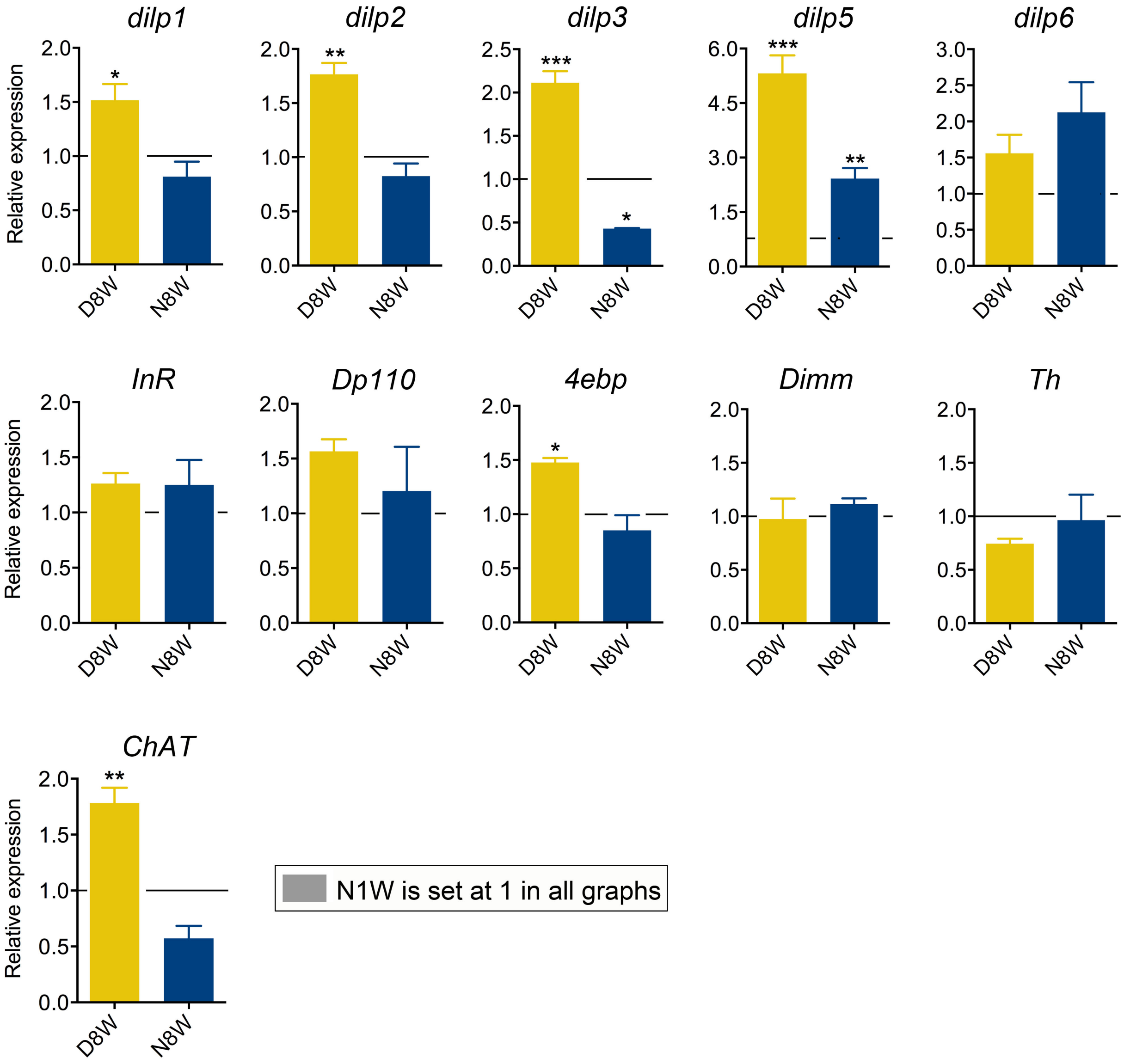

Supplementary Figure 9. Transcripts of several genes in head extracts were affected by diapause conditions, but few by aging. Four of five *Drosophila* insulin-like peptides (*dilp1, 2, 3,* and *5*) and *4ebp* increased in flies kept for 8 weeks in dormancy (D8W), whereas *dilp*6, the PI3K subunit *Dp110* and the transcription factor *Dimmed* (*Dimm*) did not change. Tyrosine hydroxylase (Th) transcript did not change, while that of choline acetyltransferase (*ChAT*) increased with diapause. Only two transcripts were affected by normal aging (N8W): *dilp3* and *dilp5*. Data are presented as means ± S.E.M, n = 3 replicates for all samples (for each biological replicate there are 2 technical ones). Each replicate consists of 20–30 heads for each genotype (*p < 0.05, **p < 0.01, ***p < 0.001, compared to N1W flies (expression set at 1), as assessed by one-way ANOVA with Tukey's multiple comparison). The horizontal lines indicate the level of N1W expression.
